# Defining the ATPome reveals cross-optimization of metabolic pathways

**DOI:** 10.1038/s41467-020-18084-6

**Published:** 2020-08-28

**Authors:** Neal K. Bennett, Mai K. Nguyen, Maxwell A. Darch, Hiroki J. Nakaoka, Derek Cousineau, Johanna ten Hoeve, Thomas G. Graeber, Markus Schuelke, Emin Maltepe, Martin Kampmann, Bryce A. Mendelsohn, Jean L. Nakamura, Ken Nakamura

**Affiliations:** 1 Gladstone Institute of Neurological Disease, San Francisco, CA 94158 USA; 2 Department of Radiation Oncology, University of California, San Francisco, CA 94158 USA; 3 UCLA Metabolomics Center, Crump Institute for Molecular Imaging, Department of Molecular and Medical Pharmacology, University of California, Los Angeles, CA 90095 USA; 4 NeuroCure Clinical Research Center, Charité–Universitätsmedizin Berlin, 10117 Berlin, Germany; 5 Department of Neuropediatrics, Charité–Universitätsmedizin Berlin, 13353 Berlin, Germany; 6 Department of Pediatrics, University of California, San Francisco, CA USA; 7 Department of Biochemistry and Biophysics and Institute for Neurodegenerative Diseases, University of California, San Francisco, CA USA; 8 Chan Zuckerberg Biohub, San Francisco, CA 94158 USA; 9 Department of Neurology, University of California, San Francisco, CA 94158 USA; 10 Graduate Program in Biomedical Sciences, University of California, San Francisco, CA USA; 11 Graduate Program in Neuroscience, University of California, San Francisco, CA USA

**Keywords:** Cell growth, Metabolic pathways, Functional genomics

## Abstract

Disrupted energy metabolism drives cell dysfunction and disease, but approaches to increase or preserve ATP are lacking. To generate a comprehensive metabolic map of genes and pathways that regulate cellular ATP—the ATPome—we conducted a genome-wide CRISPR interference/activation screen integrated with an ATP biosensor. We show that ATP level is modulated by distinct mechanisms that promote energy production or inhibit consumption. In our system HK2 is the greatest ATP consumer, indicating energy failure may not be a general deficiency in producing ATP, but rather failure to recoup the ATP cost of glycolysis and diversion of glucose metabolites to the pentose phosphate pathway. We identify systems-level reciprocal inhibition between the HIF1 pathway and mitochondria; glycolysis-promoting enzymes inhibit respiration even when there is no glycolytic ATP production, and vice versa. Consequently, suppressing alternative metabolism modes paradoxically increases energy levels under substrate restriction. This work reveals mechanisms of metabolic control, and identifies therapeutic targets to correct energy failure.

## Abstract (web summary)

Energy metabolism and ATP levels are controlled by an interlocking network of pathways. Here, the authors apply a genome-wide CRISPR screen to define genes that increase or decrease ATP levels to define the “ATPome”, a map of pathways that contribute to cellular ATP regulation.

## Introduction

ATP is the key energy-carrying molecule in all cells, and failure to maintain adequate ATP levels is critical in many diseases, from mitochondrial disorders to cancer and neurodegeneration^1–3^. ATP levels are regulated by the balance of production and consumption. In mammalian cells, ATP is generated by either mitochondrial oxidative phosphorylation or cytoplasmic glycolysis, while ATP consumption depends on the sum of cellular energy requirements. However, we understand very little about which cellular processes are most energy consuming, and there has been no systematic genome-wide assessment of which genes are most critical to regulating ATP levels.

We previously developed a high-throughput screen that uses fluorescence-activated cell sorting (FACS) and a fluorescence resonance energy transfer (FRET) ATP biosensor that we optimized to identify genes that regulate ATP levels. Analysis of a mitochondrial-gene-enriched CRISPRi sublibrary identified mitochondrial ribosomal proteins and a subset of other mitochondrial genes, as particularly critical in maintaining mitochondrial-derived ATP levels^4^. However, large gaps remain regarding which non-mitochondrial genes are critical for cellular ATP homeostasis, and it remains unresolved how ATP can be preserved in the setting of energy failure, whether ATP can be boosted to supraphysiologic levels, and if either of these strategies have therapeutic potential. Similarly, we don’t understand when and how increased ATP production contributes to tumorigenesis, which is frequently characterized by rewired glucose metabolism to generate biosynthetic precursors at the expense of ATP^5^, suggesting an excess of ATP at the opposite spectrum from energy failure.

Here, we combine our screening paradigm with CRISPRi and CRISPRa technology to screen the entire human genome for genes and pathways that regulate cellular ATP across three distinct metabolic conditions, and thereby define the cellular ATPome at the genetic level.

## Results

### A whole-genome screen for genetic regulators of ATP

**Fig. 1:**
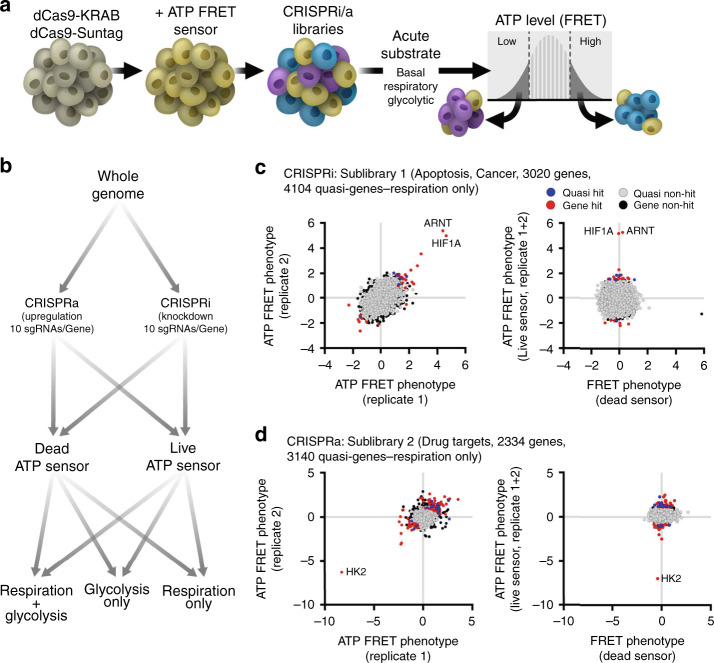
Whole-genome screens to identify genetic modifiers of ATP. **a**, **b** Schema shows single-cell detection and sorting based on ATP. K562 cells stably expressing either dCas9-KRAB (CRISPRi) or dCas9-Suntag (CRISPRa), and an ATP FRET sensor (Clover and mApple separated by an ATP-binding domain or dead sensor) were transduced with either CRISPRi (downregulation) or CRISPRa (upregulation) sgRNA sublibraries with ten sgRNAs/gene. Cells were then exposed to substrate and drug conditions forcing ATP synthesis from glycolysis only (5 μM oligo and glucose), oxidative phosphorylation only (pyruvate and 10 mM 2DG), or allowing either (basal) for 30 min prior to sorting. Cells in the lowest and highest ATP quartiles were isolated by flow cytometry, and the relative enrichment of each sgRNA was quantified by deep sequencing. Adapted from ref. ^4^. **c**, **d** Individual gene phenotypes in sample CRISPRi (**c**) and CRISPRa (**d**) sublibraries in respiratory conditions. FRET phenotype is the average of the three sgRNAs with the largest phenotypes. Graphs on left show ATP FRET phenotype of replicate 1 (*X*-axis) versus 2 (*Y-*axis). High ATP gene hits (red) show increased phenotype on both repetitions, while quasi-gene hits (blue) indicate false positives. Graphs on right show FRET phenotype for the live ATP FRET sensor (*Y*-axis) versus dead FRET sensor (*X*-axis). ATP hits do not have significantly altered FRET phenotypes with the dead sensor, indicating the specificity of the live sensor phenotype for ATP. sgRNA with large dead sensor phenotypes were excluded from analysis. Source data are provided as a Source data file.

We used a FACS-based assay we developed^4^ to screen the genome for regulators of cellular ATP levels (Fig. 1a, b). K562 cells expressing an optimized live or dead FRET-based ATP biosensor (Clover-mApple ATP or dead sensors), and either dCas9-KRAB for CRISPR inhibition (CRISPRi) or dCas9-Suntag for CRISPR activation (CRISPRa), were transduced with lentiviruses expressing corresponding CRISPRi/a sublibraries. We screened the entire genome with seven CRISPRi and seven CRISPRa sublibraries, each of which contains ten sgRNAs per gene and ~1400 nontargeting control guides^6^. We then used a fluorescent ATP biosensor and FACS to isolate cells with high and low ATP levels (Supplementary Fig. 1a), and determined the relative enrichment of each CRISPRi/a sgRNA in each pool by deep sequencing. For each screen, genes were assigned an ATP phenotype reflecting the magnitude of change of the top three sgRNAs against that gene (see “Methods” section).

The CRISPRi/a screens were performed in three different metabolic conditions. Immediately prior to FACS, cells were incubated in substrates that required that all ATP production come from (a) mitochondria (i.e., respiratory condition, 2-deoxyglucose (2DG; 10 mM) to block all glycolysis, with pyruvate as the primary metabolic substrate)^4^, (b) glycolysis (glycolytic condition, 5 μM oligomycin to block all respiration, with glucose as substrate), or (c) either substrate (basal condition). Glycolytic conditions also included a low dose of 2DG (3 mM) to reduce ATP from baseline, since ATP levels did not acutely decrease in K562 cells with oligo alone in the presence of glucose, while still enabling ATP production through glycolysis^4^. Although in standard physiological contexts cells may not be forced to rely exclusively on respiration or glycolysis, this may occur in extreme physiologic settings or in pathologic conditions, such as ischemia or cancer. Moreover, probing these conditions enabled us to identify genes that regulate cellular energy levels through diverse mechanisms and provided insight into their mechanisms of action. Notably, the respiratory and glycolytic conditions both produced a partial and stable drop in ATP levels^4^, mimicking the transient drops in ATP that may underlie much of the toxicity in mitochondrial disorders^7^. Therefore, high ATP hits help preserve normal ATP levels during metabolic stress, while producing supraphysiologic ATP levels under basal conditions.

Our previous interrogation of mitochondrial genes illustrated that our screening approach is highly specific and sensitive^4^. Consistent with this, our whole-genome screens identified multiple hits with robust ATP phenotypes that were apparent on both repetitions, and not with the dead sensor (Fig. 1c, d), and ATP phenotypes were independent of cell size (Supplementary Fig. 2a).

### Genes and pathways that regulate ATP when expressed at physiologic levels (CRISPRi)

The CRISPRi screens identified numerous gene pathways and ontologies that robustly impact ATP when knocked down. Several were expected, in particular mitochondrial translation and respiratory chain genes that were strongly enriched in low ATP hits in respiratory conditions, but increased ATP in both glycolytic and basal conditions. Roughly 80% of mitochondrial translation and 13% of respiratory chain genes in the genome were included in the mitochondrial-enriched CRISPRi sublibrary, we previously reported^4^. As expected, most (90%) of these hits were similarly identified as low ATP respiratory hits when glutamine was substituted for pyruvate, as the primary carbon source^4^ (Supplementary Fig. 2b). The current study also identified additional prominent mitochondrial genes, including the mitochondrial enzyme *HSD17B10* (Aβ-binding alcohol dehydrogenase, ABAD) involved in isoleucine and neurosteroid metabolism, which was a very strong low ATP respiratory hit and a weak high ATP glycolytic hit (Supplementary Data 1). Mutation of *HSD17B10* impairs mitochondrial RNA processing, disrupts respiratory chain complex function, and leads to neurodegeneration, cardiomyopathy, and early death^8,9^. *HSD17B10* is also upregulated in Alzheimer’s disease, and its activity is disrupted by Aβ^10^. We also identified *TMEM261* (*DMAC1*), a recently described functionally critical component of complex I (ref.^11^), as the most robust low ATP hit in a sublibrary of 1096 genes.

**Fig. 2:**
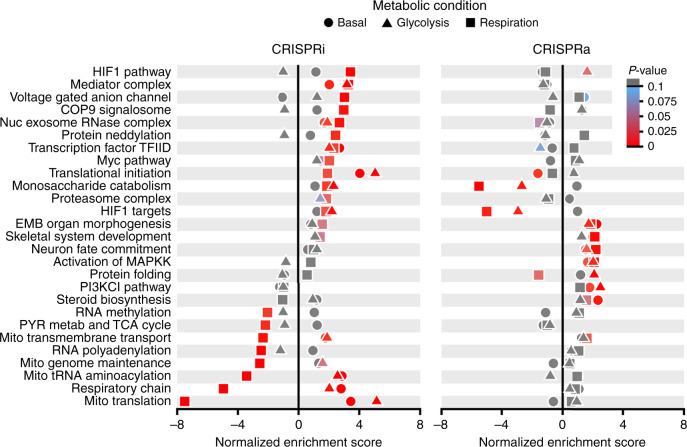
CRISPR screens identify genetic pathways that regulate ATP level. Gene set enrichment analysis on all screened genes (CRISPRi left and CRISPRa right) listed based on CRISPRi ATP phenotype in respiratory conditions, reveals enrichment of pathways and gene ontologies that regulate ATP levels under basal metabolism (circles), metabolism restricted to respiration only (squares), or glycolysis only (triangles). Significantly enriched pathways and ontologies are colored red. Pathways enriched with CRISPRa were mostly distinct from those enriched by CRISPRi. Source data are provided as a Source data file. *P*-value is estimated with GSEA software using an empirical phenotype-based permutation test. Multiple testing correction via false discovery rate estimation and exact *p*-values for each pathway are provided in Supplementary Data 2.

**Fig. 3:**
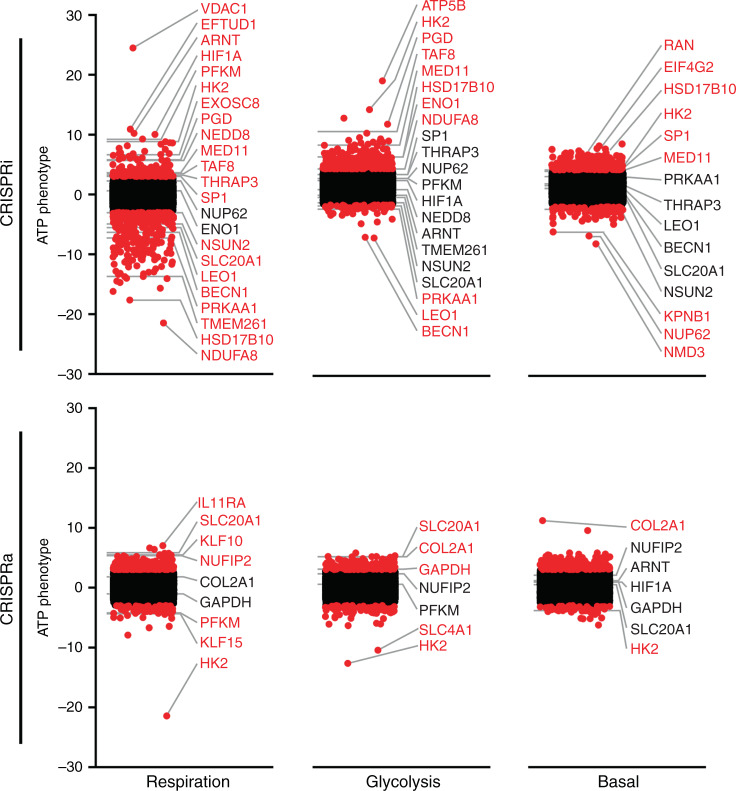
Whole-genome summary of genes that alter ATP as a function of metabolic substrate. ATP phenotypes (*Z*-scores) for each gene normalized to their respective sublibrary’s nontargeting guides, targeted with both CRISPRi and CRISPRa in respiratory, glycolytic, and basal metabolic substrates. Hits marked by red dots have phenotypes greater than three standard deviations from the mean of nontargeting guides. Labels identify genes with particularly robust phenotypes, or that are either members of notable classes or have characteristic responses to substrates discussed in the text. Source data are provided as a Source data file.

Interestingly, our CRISPRi screen highlighted the hypoxia-inducible factor-1 (HIF1) pathway, known to promote glycolysis under hypoxic conditions, as one of the strongest drivers of ATP in respiratory conditions. In contrast, CRISPRi against the HIF1 pathway (downstream transcription targets not included) had little effect on ATP levels in glycolytic conditions (Fig. 2, Supplementary Data 2). HIF1α and aryl hydrocarbon nuclear translocator (*ARNT*, HIF1ß), which form the functional HIF1 molecule, were the two most prominent high ATP genes in a sublibrary of 3020 genes (Fig. 1c). Suppression of glycolytic genes regulated by HIF1, such as *PGK1*, *SLC2A1*, and hexokinase 2 (*HK2*; represented in both the monosaccharide catabolism ontology and the downstream HIF1 targets pathway), as well as upstream genes that regulate HIF1 (e.g., *CHCHD4* (ref.^12^)), and HIF1 stability, such as the COP9 signalosome (e.g., *COPS4* and *COPS8*) and neddylation (e.g., *NEDD8*)^13^, also increased ATP levels in respiration-only conditions. Suppression of HK2 binding partner VDAC1 (ref. ^14^) also strongly increased ATP under respiratory conditions (Fig. 3), suggesting a pivotal role for HK2–VDAC1 interaction in ATP regulation. Our findings unify these hits as a substrate-specific network with ARNT and HIF1α at the center. Therefore, cells maintain residual cross-inhibitory metabolic activity between respiratory and glycolytic pathways, and suppressing a subset of respiratory or glycolytic genes reciprocally activates the other, with this bioenergetic activation persisting even when the pathway being suppressed is not actively producing ATP.

There were far fewer low ATP hits in glycolytic and basal conditions (72 and 93 genes, respectively, compared to 345 in respiratory conditions). As such, our whole-genome analysis reveals that there are more respiratory than glycolytic genes that control ATP, perhaps reflecting the overall importance, complexity, and tunability of respiration for ATP homeostasis.

A small number of high ATP CRISPRi hits increased ATP across all three substrate conditions. These included genes that regulate transcription, such as the mediator complex (e.g., *MED1* and *MED11*), or regulate RNA and protein quality control, such as the nuclear exosome RNAse complex (e.g., *EXOSC8*), the proteasome complex, and translational initiation pathways, as well as *SP1*, which regulates cell growth, apoptosis, and chromatin remodeling^15^, and *HK2*. Conversely, a subset of hits involved in RNA processing decreased ATP across multiple substrates, including the RNA polymerase-associated protein Leo1 (ref. ^16^) in respiratory and glycolytic conditions, and *NSUN2* (RNA methylation), which was a weak hit across all three substrates. However, in the entire genome, there were no robust low ATP hits across all substrates, perhaps reflecting the redundancy of protective mechanisms that maintain ATP.

### Genes that regulate ATP levels upon overexpression (CRISPRa)

The CRISPRa screens revealed fewer genes impacting ATP levels in any substrate than the corresponding CRISPRi screens (793 for CRISPRa across all substrates, 1250 for CRISPRi), suggesting that increasing gene expression is less likely to impact ATP levels than decreasing expression. Notably, many of the enriched pathways were distinct from those enriched by CRISPRi (Fig. 2, Supplementary Data 2). In particular, upregulation of several developmental pathways, including embryonic organ morphogenesis, skeletal system development, and neuron fate commitment increased ATP, suggesting that energy levels may be regulated in coordination with development across a wide range of paradigms. Other pathways enriched among high ATP hits included steroid biosynthesis, and the PI3KCI pathway in basal conditions. The PI3KCI pathway was also enriched with high ATP genes in glycolytic conditions, as were the protein folding pathway and MAPKK activation ontology. Steroid biogenesis and PI3K signaling comprise important pathways in development and regulation of metabolism^17,18^, and steroid signaling can co-activate PI3K signaling^19^.

Beyond these pathways, individual CRISPRa hits included a diverse range of genes. The sodium–phosphate symporter *SLC20A1* (PiT1), which increases ATP and supports chondrogenesis by promoting phosphate uptake^20^, was among the strongest high ATP hits in both respiratory and glycolytic conditions (Fig. 3). Also prominent was *COL2A1* (collagen type II alpha-1), mutation of which produces type II collagenopathies^21^, but which has no known link to energy metabolism. *COL2A1* was among the strongest CRISPRa high ATP hits in glycolytic conditions, and the strongest in basal conditions. Other notable CRISPRa hits included nuclear fragile X mental retardation-interacting protein 2 (NUFIP2) and the zinc finger protein KLF10, which increased ATP specifically in respiratory conditions.

Increasing expression of numerous genes also decreased ATP (i.e., CRISPRa low ATP hits). In particular, increasing the glycolysis-associated HIF1 target and monosaccharide catabolism ontologies decreased ATP under respiratory and glycolytic conditions, driven especially by HK2, which was present in both. Indeed, HK2 was the single strongest driver of low ATP levels in the genome in both respiratory and glycolytic conditions, and was also a low ATP hit in basal conditions in our CRISPRa screen. Other prominent low ATP hits included the transcriptional regulator KLF15 and the anion exchanger SLC4A1, indicating that genes within these classes regulate ATP in both directions.

### CRISPRi–CRISPRa concordance

Most high ATP hits in CRISPRi were not low ATP hits in CRISPRa, and vice versa. This suggests that in general, increasing expression of genes involved in energy production (e.g., respiratory chain genes) does not further increase ATP production. However, there were examples where CRISPRi and CRISPRa changed in opposing directions in the same substrate. In particular, as discussed, *HK2* was a high ATP CRISPRi hit and a low ATP CRISPRa hit, in respiratory, glycolytic, and basal conditions (Fig. 3), indicating that HK2 regulates ATP levels across a wide dynamic range. Many genes in the HIF1 pathway, including *HIF1A* and *ARNT*, also had inverse phenotypes (high ATP by CRISPRi and low ATP by CRISPRa), although most of these did not achieve significance as individual hits. Conversely, *SLC20A1* was a low ATP hit in CRISPRi respiratory conditions and borderline low in CRISPRi glycolytic conditions, but a high ATP hit in CRISPRa respiratory and glycolytic conditions.

### High ATP hits boost aerobic respiration through distinct mechanisms

To gain insight into how high ATP hits increase ATP, we generated both K562 and immortalized human fibroblast cell lines expressing individual sgRNAs against a subset of ATP hits identified in the main screen, and confirmed knockdown or overexpression (Supplementary Fig. 3a–c). Using luciferase assay as a second ATP readout (Supplementary Fig. 3d), we confirmed that robust CRISPRi and CRISPRa high ATP hits preserved ATP, and low ATP hits decreased it (Supplementary Fig. 3e–g). However, consistent with our prior findings^4^, the luciferase assay was less sensitive than the FRET assay, presumably because cells were pooled for luciferase analysis rather than analyzed individually, as in the FRET assay.

**Fig. 4:**
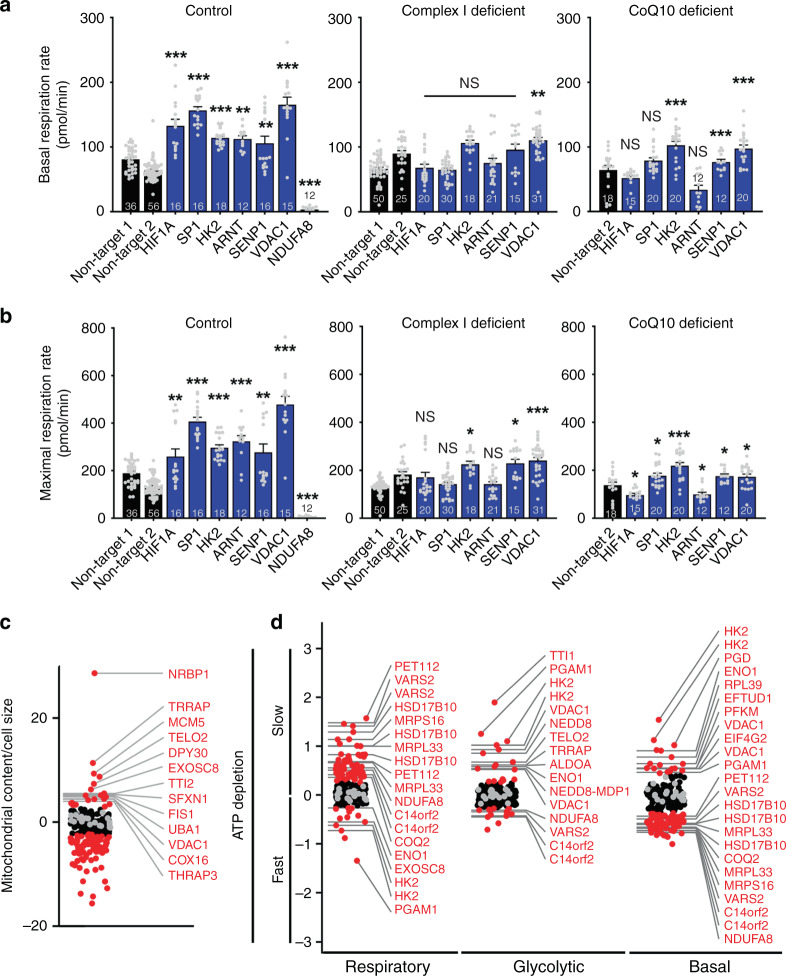
High ATP hits increase ATP through distinct mechanisms. **a**, **b** Subset of high ATP respiratory hits require an intact respiratory chain to boost respiration. Basal (**a**) and maximal respiration (pmol O_2_/min) (**b**) of fibroblast lines from control and respiratory chain deficient patients, measured with a Seahorse Analyzer after the addition of the mitochondrial uncoupler FCCP (1 μM). All data were analyzed from the total number of samples shown in the bars, in at least two experiments per line. Data are presented as mean values ± SEM; *p < 0.05, **p < 0.01, ***p < 0.001, NS not significant by one-way ANOVA with Dunnett multiple comparisons test. **c** Subset of ATP respiratory hits increase mitochondrial content. K562 cells expressing the CRISPRi mini-library enriched in ATP respiratory hits. Data show sgRNAs that increased mitochondrial content (MitoTracker Green) normalized to cell size (forward scatter) by FACS. Hits marked by red dots have phenotypes greater than three standard deviations from the mean of nontargeting guides. *n* = 4 replicates of 500k collected cells. **d** K562 cells expressing the mini-library of ATP respiratory hits were preincubated for 1 h in either respiratory, glycolytic, or basal conditions. The rate of ATP consumption was then estimated as the rate of ATP phenotype decline relative to nontargeting guides when all ATP production was blocked for 16 min (10 mM 2DG, 1 mM IAA, and 5 μM oligo). Gene knockdowns that decrease ATP consumption have greater enrichment in high ATP fractions after ATP production is blocked. Hits marked by red dots have phenotypes greater than three standard deviations from the mean of nontargeting guides. *n* = 2 replicates of 150k cells for each pre- and post-ATP depletion group. Source data and exact *p*-values are provided as a Source data file.

We then assessed the impact of knocking down the CRISPRi hits *HIF1A* and *ARNT*, the HIF1 targets *HK2* and *VDAC1* (ref. ^22^), and the HIF1-regulating proteins SENP1 and SP1 (ref. ^23^), on respiratory function in the fibroblast lines. Knockdown of these CRISPRi high ATP hits increased both basal and maximal respiration respiration (Fig. 4a, b), assessed on a Seahorse Analyzer. In contrast, knocking down the strong low ATP respiratory hit NDUFA8 (a complex I subunit) essentially abolished respiration.

We next asked if these same high ATP hits preserve ATP levels in cells with genetic defects in cellular respiration. Knockdown of *HK2*, *SENP1*, or *VDAC1*-boosted basal and/or maximal respiration in fibroblasts generated from patients with two distinct forms of Leigh syndrome (mutations in the complex I subunit *NDUFS4*, and in the COQ10 biosynthetic enzyme *PDSS2* (ref. ^24^), Fig. 4a, b, Supplementary Fig. 4a–d), although the extent of increase was less than in wild-type cells. Moreover, knockdown of *ARNT* or *HIF1A* did not increase basal or maximal respiration on either of these backgrounds, despite increasing respiration on the wild-type background, suggesting that the mechanism of increased ATP production requires an intact respiratory chain. Although *HK2* is a downstream target of HIF1, these data indicate that our high ATP hits act through distinct mechanisms, and the efficacy of ATP increasing therapies will depend on matching the strategy to the specific insult.

Increased respiration could result from increased substrate delivery, mitochondrial content, or respiratory capacity. To determine if high ATP hits increased mitochondrial content, we transduced K562 cells with mini-library, including 1–3 of the strongest sgRNAs for each of 150 genes enriched in high and low ATP respiratory conditions (Fig. 4c, Supplementary Fig. 1b, Supplementary Data 3), and sorted cells based on mitochondrial content (MitoTracker green) normalized to cell size (forward scatter^25^). We identified high ATP respiratory hits that increase mitochondrial content, including *NRBP1*, *TELO2*, *EXOSC8*, and *VDAC1*, the latter of that was previously implicated in mitophagy^26^. We validated their effects with individual knockdowns (Supplementary Fig. 4e). These data suggest a subset of high ATP genes increase ATP by increasing mitochondrial content.

### ATP consumption

The rate of ATP consumption is a determinant of cellular ATP levels, but there has been no systematic assessment of which cellular processes are most energy consuming or which genes are most critical to regulating consumptive processes. As such, we asked if our high ATP hits decrease ATP consumption. We reasoned that when ATP production is blocked, ATP levels will decline more slowly in cells that consume less energy, assessed with the ATP FRET sensor (Fig. 4d, Supplementary Data 3). Cells expressing the mini-library were preincubated in either respiratory, glycolytic, or basal conditions. We then acutely blocked all ATP production for 10–15 min (with oligo, 2DG, and the GAPDH inhibitor iodoacetate, IA), and determined the change in ATP phenotype, as a surrogate for the rate of ATP consumption. In respiratory conditions, knockdown of numerous mitochondrial genes that were low ATP respiratory hits (e.g., *VARS2*, *MRPL33*, and *HSD17B10*) reduced energy consumption, likely reflecting a compensatory response to their inability to produce energy in respiratory conditions. In contrast, in basal and glycolytic conditions, there was an enrichment in glycolytic enzymes, including *HK2* and *ENO1*, among genes that decreased energy consumption, while knockdown of mitochondrial genes increased energy consumption.

To gain insight into how a single gene product might alter energy consumption sufficiently to influence cellular ATP levels, we focused on *HK2*. As *HK2* metabolism of both 2DG and glucose requires ATP^27^, we asked if their metabolism serves as the ATP sink. First, we preincubated cells in either respiratory, glycolytic, or basal conditions, then blocked all ATP production with oligo and 2DG (without IA; Supplementary Fig. 5a). In this setting, blocking protein synthesis with cycloheximide decreased ATP consumption in all three conditions, while knockdown of the low ATP CRISPRi hit *NDUFA8* decreased ATP consumption only in respiratory conditions. *HK2* knockdown decreased ATP consumption in all three conditions, including respiratory conditions in the absence of glucose, and basal conditions in the absence of 2DG, indicating that HK2-mediated phosphorylation of 2DG or glucose is a major source of energy consumption. In contrast, knockdown of *HIF1A* had no impact on ATP consumption.

Although 2DG-6-phosphate can’t be metabolized further, glucose-6-phosphate can be metabolized through glycolysis to replenish ATP lost during its production. To confirm that glucose metabolism by HK2 depletes ATP, we blocked ATP production with oligomycin and IA (without 2DG) to block glycolysis at a distal step from hexokinase. *HK2* knockdown still decreased energy consumption in cells preincubated with glucose in basal and glycolytic conditions, but now actually increased the rate of ATP consumption in respiratory conditions, where glucose was absent (Supplementary Fig. 5b).

### Hexokinase metabolomics

**Fig. 5:**
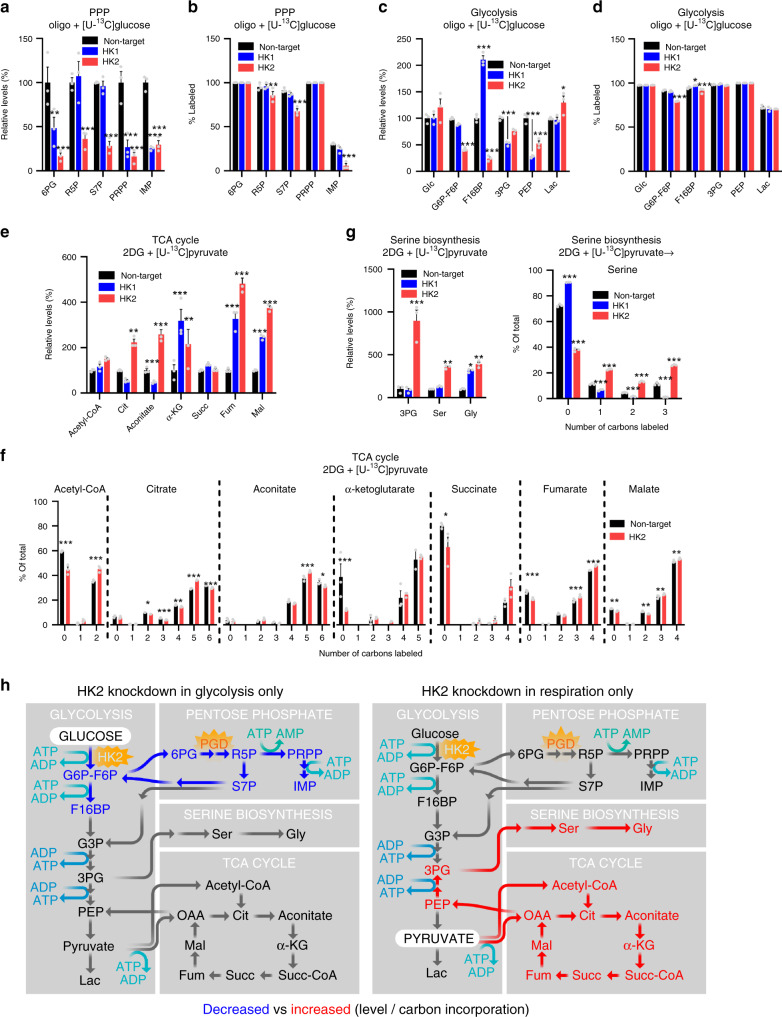
HK2 preferentially increases PPP, and decreases TCA and serine metabolites. **a**–**d** K562 cells were incubated for 2 h with glycolysis-only substrates (2 mM [U-^13^C]glucose, 5 μM oligo, and low dose 2DG (3 mM)). **a** *HK2* knockdown decreased the size of PPP metabolite pools, while *HK1* knockdown had variable effects. **b** *HK2* knockdown also decreased incorporation of glucose carbons into PPP metabolite ribose 5-phophate and sedoheptulose 7-phosphate, while *HK1* knockdown did not. **c** *HK2* and *HK1* knockdown had variable effects on lowering the level of glycolytic metabolites, and **d** did not affect the incorporation of glucose carbons into into late glycolytic metabolites. **e**–**g** Cells were incubated with respiration-only substrates (10 mM [U-^13^C]pyruvate and 10 mM 2DG) for 2 h (**e**, **g**) or 6 h (**f**). **e** *HK2* knockdown increased the size of most TCA cycle metabolite pools, while *HK1* knockdown had variable effects. **f** *HK2* knockdown increased the incorporation of pyruvate carbons into TCA metabolites. **g** *HK2* knockdown also increased the size and incorporation of pyruvate carbons into serine metabolite pools, while *HK1* knockdown did not. For **a**–**g**, *n* = 3 samples of 500k cells, *p < 0.05, **p < 0.01, ***p < 0.001 versus nontargeting control, two-way ANOVA with Sidak’s multiple comparisons test. **h** Schematic of hypothesized key metabolic pathways perturbed with *HK2* knockdown, when either glucose (left) or pyruvate (right) is the primary metabolic substrate, with red or blue indicating increases or decreases, respectively, in level or carbon incorporation. Data are presented as mean values ± SEM. Source data and exact *p*-values are provided as a Source data file.

Our data support that blocking HK2 phosphorylation of either glucose or 2DG increases ATP levels by decreasing ATP consumption, and the observation of ATP depletion with 2DG indicates that a failure to recoup the initial ATP investment required for glucose phosphorylation can drive ATP depletion. In addition, the finding that HK2 also increases ATP consumption with glucose alone as substrate (Fig. 4d, Supplementary Fig. 5a, b) indicates that HK2 shifts the pattern of glucose metabolism toward more energy consuming versus producing pathways. Indeed, under glycolytic conditions, *HK2* knockdown markedly decreased total levels of PPP metabolites (Fig. 5a), as well as the amount of glucose-derived carbon incorporated into these metabolite pools during 2 h of labeling (Fig. 5b), while *HK1* knockdown resulted in either a smaller or no effect on the PPP metabolites.

Importantly, the PPP enzyme PGD was also both a high ATP CRISPRi hit in glycolytic conditions and a slow ATP consumer under basal conditions (Figs. 3 and 4d), further highlighting the PPP and its downstream pathways as major energy consumers.

*HK2* knockdown also decreased the amount of most glycolysis metabolites under glycolytic conditions (Fig. 5c), and did not affect glucose-derived carbon incorporated into most late glycolysis pathway metabolites (Fig. 5d). As such, our data indicate that increased glycolytic ATP with *HK2* knockdown results from decreased ATP consumption with diversion of glucose metabolites through the anabolic PPP pathway, not increased ATP production.

Interestingly, we also found that *HK2* knockdown increased the net amount of pyruvate-derived carbon incorporated into TCA cycle metabolite pools in respiratory conditions (where pyruvate is the substrate with glycolysis fully blocked, Fig. 5e, f), indicating that HK2 also impacts downstream TCA metabolites independent of its effects on pyruvate generation through glycolysis. These results presumably explain why *HK2* knockdown also increases aerobic respiration (Fig. 4a, b).

Notably, *HK2* knockdown also increased levels of serine, consistent with prior observations^28^. Interestingly, we found this was converted from pyruvate (Fig. 5g, h, Supplementary Fig. 5c–e), rather than being synthesized from glucose (Supplementary Fig. 5c, d), while *HK1* knockdown did not increase pyruvate conversion to serine. These results support that under respiratory conditions, pyruvate is converted to serine via oxaloacetate (an ATP-requiring step), likely at least in part explaining why *HK2* knockdown increased ATP consumption under respiratory conditions. The increase in serine biosynthesis and downstream folate cycle can be a source of NADPH comparable to the PPP, and therefore may be compensatory to maintain redox balance^29^.

### Impact of ATP on growth and survival

When energy production decreases, cells may compensate by decreasing growth and proliferation, while increased energy levels may accelerate those processes. However, other functions of the respiratory chain also influence proliferation and growth^30^, raising fundamental questions about how and when the ATP level governs growth, and which genes mediate this functional connection. In solid tumors, cancer cells must overcome metabolic challenges related to growth in diverse microenvironments and intratumoral hypoxia^31^. To test the effects of changing ATP levels on growth in a solid tumor cell line, we transduced HCC827 human lung cancer cells expressing dCas9-KRAB with the mini-library enriched in CRISPRi respiratory hits, and determined the impact of each sgRNA on growth in different metabolic conditions.

Under respiratory conditions, knockdown of most mitochondrial ribosomal and other mitochondrial proteins that decrease mitochondrial-derived ATP produced a significant albeit modest negative impact on cell growth (Supplementary Fig. 6a, Supplementary Data 4). This suggests that the tumor cells require mitochondrial-derived ATP under these conditions, although they maintained sufficient ATP to support growth even when mitochondrial-derived ATP was limited, likely because sufficient glucose (11.1 mM) was metabolized despite competitive inhibition by 10 mM 2DG.

**Fig. 6:**
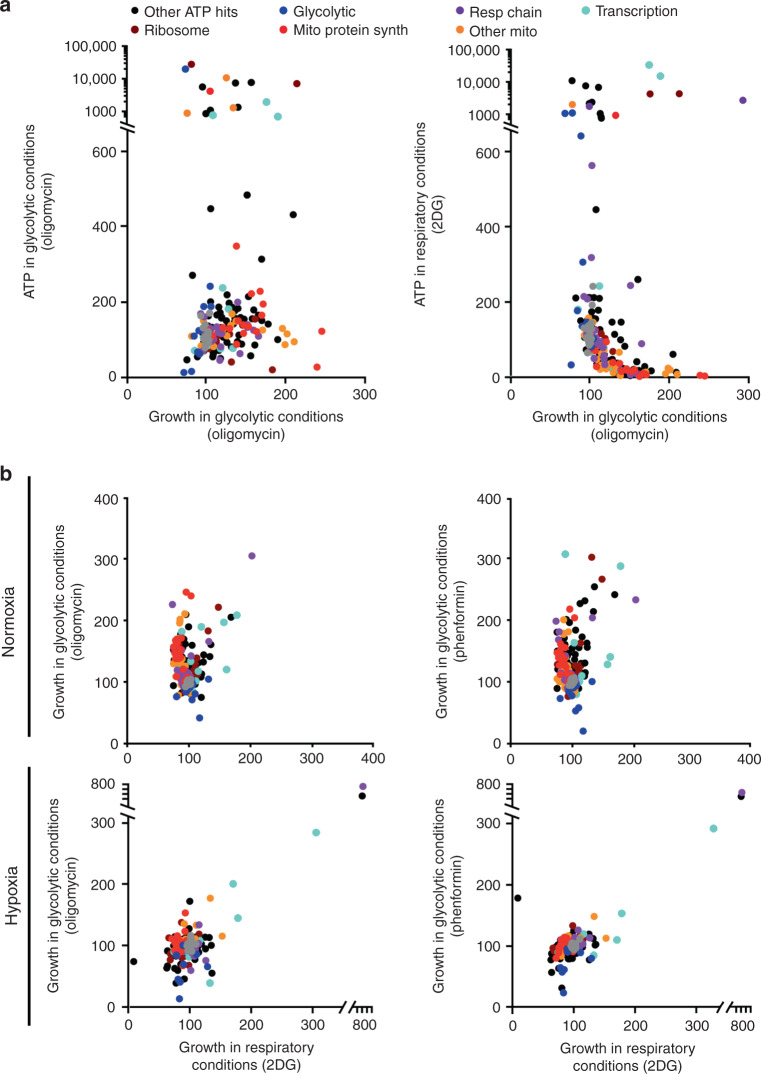
High ATP hits increase growth through ATP-dependent and independent mechanisms. **a** HCC827 cells expressing the CRISPRi mini-library were grown for 3 days in glycolytic conditions (5 μM oligo), and the fold-impact of each sgRNA on growth (mean read count, normalized to nontargeting controls) plotted versus ATP level measured in parallel experiments. Data from four replicates of 1 M collected cells per group for ATP, six replicates of 1 M collected cells per group for growth. For growth and ATP in glycolytic conditions, nontargeting guides Pearson *r* = −0.162, *n* = 19, *p* = 0.510; all targeting guides *r* = 0.361, *n* = 185, *p* < 0.001; glycolytic *r* = 0.796, *n* = 9, *p* < 0.05; mitochondrial ribosome *r* = 0.600, *n* = 14, *p* = 0.0234, *p*-values for nonzero slope or null hypothesis of no correlation by Pearson’s correlation test after removing outliers with ROUT method. **b** HCC827 cells expressing the CRISPRi mini-library were grown for 3 days in either glycolytic (5 μM oligo or 0.5 mM phenformin) or respiratory (10 mM 2DG) substrates, both in normoxia (top) and hypoxia (1% O_2_, bottom), and the impact of metabolic substrate on growth compared. In normoxia, knockdown of 48/77 and 40/78 mitochondrial genes increased growth >3 SD from nontargeting guides in oligo and phenformin, respectively, while only 3/78 did for either substrate in hypoxia. *n* = 3 replicates of 1 M collected cells per group for growth in hypoxia, and *n* = 6 replicates of 1 M collected cells per group for growth in normoxia. Source data, exact *p*-values, and 95% confidence intervals are provided as a Source data file.

Indeed, low glycolytic-derived ATP was strongly correlated with decreased growth in glycolytic conditions in HCC827 cells (Fig. 6a, Pearson *r* = 0.796, *p* < 0.05; nontargeting *r* = 0.162, *p* = 0.510). In particular, decreasing ARNT both markedly decreased glycolytic-derived ATP and growth in glycolytic conditions (Supplementary Fig. 6a, b). Although decreased ARNT might impair growth through ATP-independent mechanisms, knocking down ARNT in respiratory conditions (which increases ATP, Supplementary Fig. 6c) slightly increased (rather than decreased) growth (Supplementary Fig. 6b), strongly suggesting a critical role for ATP. In addition, increasing glycolytic-derived ATP increased growth in glycolytic conditions. Silencing mitochondrial genes, which increases glycolysis-derived ATP (Supplementary Fig. 6a, b)^4^, also strongly and almost uniformly promoted tumor growth under glycolytic conditions (Fig. 6a, Supplementary Data 4). However, ATP hits that more robustly increased ATP levels beyond a threshold failed to further increase the cell growth in either respiratory or glycolytic conditions (Fig. 6a, Supplementary Fig. 6a), presumably indicating that factors other than ATP limit additional growth. We hypothesize that HCC827 cells, which are transformed, have more restricted capacity for further growth than untransformed cells.

Notably, the relative growth advantage conferred by mitochondrial gene knockdown in glycolytic conditions was abrogated by hypoxia (Fig. 6b, Supplementary Fig. 6d), likely because hypoxia both represses respiration via HIF1 to favor glycolysis and also decreases ATP consumption^32^.

Concordant effects on ATP and growth were also observed after knockdown of several non-mitochondrial and non-glycolytic genes, such as *EIF2B3* and *DPY30*, which markedly and selectively increased both ATP and growth in glycolytic conditions (Supplementary Data 4).

## Discussion

Insufficient energy to support normal function can lead to neurodegenerative diseases, ischemia, and heart failure, whereas an imbalance in bioenergetic flux between glycolysis and respiration may contribute to cancer. However, there are only anecdotal examples of how to increase or preserve cellular ATP, and many genes and pathways that regulate energy levels remain to be identified. To begin to address this, we used a high-throughput FACS-based assay with an optimized FRET-based ATP sensor to screen the entire genome, with both CRISPRi and CRISPRa libraries under discrete substrate conditions. Using this approach, we defined an ATPome, identifying genes and pathways that boost or preserve ATP levels, including many genes with no previously described role in energy metabolism.

Our screening approach identifies genes that regulate ATP with high specificity and sensitivity. Nonetheless, some genes that modulate ATP were undoubtedly overlooked. First, although we assessed ten sgRNAs per gene, and knockdown with the top guides by CRISPRi was typically ≥80–90% in K562 cells^4^ (Supplementary Fig. 3a), some genes may require complete knockdown to impact ATP. Similarly, with CRISPRa, although induction is typically more than fivefold (Supplementary Fig. 3b), there were likely genes where we failed to achieve sufficient overexpression to impact ATP. In addition, some genes may impact ATP only in the context of specific energy substrates, only in specific subcellular compartments (e.g., axons), or only in specific cell types. The energy substrates we examine enable us to identify the widest possible range of ATP-regulating genes and pathways; however, we cannot exclude the possibility that a small number of our hits were influenced by other effects of 2DG or oligomycin independent of their effects on respiration and glycolysis. However, we expect that most genes we identified will similarly modulate ATP levels across cell types, as we observed concordance between two distinct cancer cell lines. While different cell types may preferentially metabolize different energy substrates when given the choice, we hypothesize that most core energy genes and pathways are shared, and hence that most cells have similar bioenergetic functions when forced to rely on the same substrate. Indeed, many of the genes we identified are critical to the function and survival of primary cell types that are more susceptible to energy failure (e.g., neurons or cardiomyocytes), including genes implicated in degeneration through energy failure^4^, highlighting the broad disease relevance of our findings.

Some changes in ATP might reflect a change in total adenine nucleotide pool size^33^, rather than a specific change in energy charge ([ATP] + 0.5[ADP]/[ATP] + [ADP] + [AMP]) regulating enzymatic function^34^. However, key genes mediating nucleotide biosynthesis or interconversion of adenine nucleotides, such as adenylate kinase (ADK) and AMP deaminases (*AMPD*) 1–3 were not identified as hits, suggesting these processes do not contribute significantly to changes in ATP in our paradigm. Nonetheless, the impact of distinct ATP-modulating genes on energy charge, and the relationship to changes in ATP level, is an important area for future investigation.

**Fig. 7:**
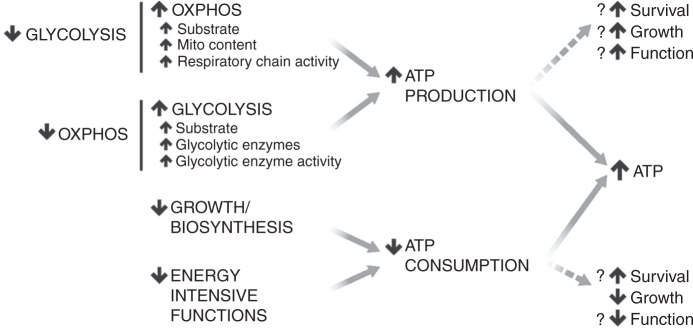
Mechanisms by which cells can increase ATP and the potential consequences. ATP levels may increase due to increased ATP production or decreased consumption. Increased production may result from either increased oxidative phosphorylation (OXPHOS) or glycolysis, and inhibiting either of these processes stimulates the other. The impact of increased ATP on cellular functions, growth, and survival likely depends on the mechanism by which ATP is increased, in addition to the specific cell type.

Our data provide important insight into the mechanisms of energy production. In mammalian cells, ATP is produced either by oxidative phosphorylation or glycolysis, with the amount of ATP produced depending on the availability of energy substrates, as well as the rate, efficiency, and total amount of enzymes supporting these processes (Fig. 7). Most ATP hits likely impact one or more of these critical aspects of ATP production. Mitochondrial ribosomal proteins and transcription factors, which comprised some of the most robust hits, influence the expression of proteins involved in energy production. Importantly, most genes that preserved ATP did so only when cells were forced to use either respiration or glycolysis. For instance, knocking down many mitochondrial ribosomal proteins decreased ATP under respiratory conditions, but increased ATP under glycolytic conditions. Therefore, an intact respiratory pathway inhibits the glycolytic ATP production, and disrupting this pathway can actually increase ATP levels under glycolytic and even basal conditions, perhaps explaining how mild hypoxia can protect mice with a respiratory chain defect^35^.

Importantly, this functional balance between glycolysis and oxidative phosphorylation is bidirectional, as inhibiting the glycolysis-promoting HIF1 pathway boosted mitochondrial-derived ATP, and this effect was phenocopied by inhibiting glycolytic enzymes, including HK2, PFKM, and ENO2 (Fig. 3, Supplementary Data 1). Similar to HIF1 regulation of respiration by limiting pyruvate availability^36^, *HK2* knockdown increased the net amount of pyruvate-derived carbon incorporated into TCA cycle metabolite pools (Fig. 5e, f). This increase in substrate for respiration likely contributes to increased basal and maximal respiration (Fig. 4a, b). The fact that mitochondrial-derived ATP can be triggered by inhibiting glycolysis at multiple steps in the glycolytic pathway suggests that a glycolytic end product(s) normally suppresses respiration and/or stimulates energy consumption.

Although inhibiting several glycolytic genes robustly boosted mitochondrial-derived ATP, there was little effect on glycolysis-derived ATP. Indeed, no glycolytic genes were robust low ATP hits in glycolytic conditions, possibly because multiple isoforms of key glycolytic genes may compensate for loss of one (e.g., *HK1–3* and *PKM1,2*). Indeed, knockdown of *HK2* (but not *HK1*) and *PGD* actually increased glycolytic ATP, without increasing the size or glucose carbon incorporation into glycolytic metabolite pools, presumably by decreasing ATP consumption rather than increasing glycolytic ATP production.

These data indicate that suppressing alternative modes of metabolism can paradoxically increase energy levels when nutrient availability is restricted, pointing to the existence of robust feedback mechanisms between low ATP, and both energy production (glycolysis and respiration) and consumption. In rare cases, this process occurred even under nutrient replete conditions. For instance, knockdown of some respiratory genes increased ATP in basal conditions, when both respiration and glycolysis were enabled. These examples by which metabolic pathways cross-optimize to increase energy may also represent distinct strategies by which energy levels could be boosted therapeutically.

A subset of hits increase ATP levels by decreasing consumption. The rate of ATP consumption depends on the summation of cellular energy requirements, including biosynthesis of molecules needed for cell growth, protein and organelle quality control, and maintenance of cellular homeostasis and functions including ionic gradients. However, while protein, RNA, and DNA synthesis have been identified as major consumers of ATP in the cell^37^, and many kinases and other processes are known to require ATP, the relative contribution of different proteins to energy consumption has been unknown. Our screen provides a systematic assessment of genes that regulate energy consumption.

We identified several genes with known roles in biosynthesis as major energy consumers, including HK2 and the PPP enzyme PGD^38,39^. Notably, HK2 was previously identified as an ATP consumer^40^ and, in our paradigm, it was the genome’s single most dynamic regulator of ATP, acting across a wide range of expression (CRISPRi and CRISPRa), and across multiple substrates. Further experiments will be required to determine if HK2 plays an equally prominent role in driving ATP consumption in other cell types and metabolic conditions. *HK2* (but not *HK1*) knockdown increased glycolytic ATP by decreasing ATP consumption, and increased glycolytic ATP may result from decreased flux through the PPP, thus decreasing energy-intensive processes, such as cell growth^41^. Moreover, HK2 binds to VDAC1 (both CRISPRi respiratory high ATP hits, and drivers of glycolytic and basal ATP consumption), enabling privileged, dynamic access to mitochondrial ATP^42^ that supports glucose phosphorylation by hexokinase^27,40,42^. Although this ATP drain may normally be replenished by subsequent steps in glycolysis and oxidative phosphorylation, use of glucose analogs, such as 2DG that cannot be metabolized by glycolysis significantly decreases ATP. Therefore, although energy failure has been almost universally viewed as a failure to produce sufficient ATP, our work demonstrates that it can result from a diversion of glucose to the PPP or a specific failure to remake the ATP spent as the initial investment in glycolysis, and that the extent of energy consumption depends heavily on energy substrate. However, many questions remain about how exactly glycolytic enzymes impact ATP levels, and indeed inhibiting several downstream glycolytic enzymes, including ENO1, PGAM1, and ALDOA, paradoxically increased glycolysis-derived ATP levels in glycolytic conditions.

We identified numerous genes without previously defined roles in regulating ATP, broadly categorized into those that increase ATP levels (CRISPRi low ATP hits and CRISPRa high ATP hits) and those that decrease or repress ATP levels (CRISPRi high ATP hits and CRISPRa low ATP hits). In most cases, changes in gene expression impacted ATP only in one direction, more commonly CRISPRi than CRISPRa, presumably because it is usually easier to disrupt the energy production (e.g., by knockdown of a key respiratory protein) than to optimize it by increasing expression. However, there are exceptions. For instance, increasing expression of glycolytic enzyme GAPDH, one of the few genes previously shown to increase ATP levels in mammalian cells^43^, increased ATP specifically in glycolytic conditions. Among genes not previously linked to energy metabolism, several classes were particularly enriched, such as transcription factors and solute carriers.

Transcription factors regulate both energy producing and consuming processes, and our data support that the process of transcription itself is energetically demanding^37^. Indeed, many genes that regulate RNA processing and transcriptional machinery modulated cellular ATP, despite lacking known relationship to defined energy pathways. For instance, knockdown of multiple components of both the transcription factor TFIID, a part of the RNA polymerase II preinitiation complex (e.g., TAF2, 4, 5, and 8), and the mediator complex, a coactivator in the transcriptional machinery that interacts with RNA polymerase II, increased ATP across multiple substrates.

Notably, knockdown of pathways connected to transcription and translation increased respiratory ATP, including the exosome RNAse complex (e.g., EXOSC8)^44^, and the RNA degradation-promoting protein NUFIP2 (ref. ^45^). Conversely, knockdown of pathways involved in RNA processing and transport, including components of the Paf1 complex (PAF1 and LEO1)^46^, and RNA methylation (e.g., NSUN2) decreased respiratory ATP. Taken together with the observation that *EXOSC8* knockdown resulted in increased respiratory ATP and mitochondrial content, our findings suggest that RNA trafficking plays an important role in regulating mitochondrial function.

While many hits impact general steps in transcription and RNA metabolism, others impacted specific transcription pathways, usually in a substrate-specific manner. The HIF1 pathway was the most robust example, impacting ATP specifically in respiratory conditions. The transcriptional repressor KLF10 was one of the strongest CRISPRa high ATP hits in respiratory conditions, and the transcriptional regulator *KLF15* was a strong CRISPRa low ATP hit. *KLF10* and *KLF15* share opposing roles in circadian clock regulation of glucose and lipid metabolism, with *KLF10* KO mice being hyperglycemic and *KLF15* KO mice hypoglycemic^47,48^, and their opposing effects on ATP may provide insights into how energy metabolism can be rapidly regulated in vivo.

A number of solute carriers had robust effects on ATP levels (22 in CRISPRi and 29 in CRISPRa), including several that transport key energy substrates. These included the high ATP CRISPRa hit sodium–phosphate symporter SLC20A1 (PiT1) and the low ATP CRISPRi hit mitochondrial phosphate carrier SLC25A3, suggesting that phosphate delivery to the mitochondria is critical to maintain ATP levels.

Another CRISPRi low ATP respiratory hit was the dicarboxylate carrier DIC (SLC25A10), which is required for the translocation of malonate, malate, and succinate across the mitochondrial inner membrane, without that cells must rely on glutamine for growth^49^. Conversely, increased expression of the glutamine transporter SLC38A3 (SNAT3), which cotransports glutamine and sodium ions in exchange for protons^50^, increased ATP in respiratory conditions, and mice lacking SLC38A3 are hypoglycemic^51^. The glucose transporter SLC2A1 (GLUT1) was a low ATP CRISPRi hit in glycolytic conditions, and inhibiting SLC2A1 can decrease glycolysis and tumor growth^52^. Overexpression of SLC16A1, a monocarboxylate transporter (MCT1) that mediates the movement of the respiratory substrates lactate and pyruvate across the plasma membrane^53^, decreased ATP in glycolytic conditions, suggesting that boosting respiratory substrate delivery inhibits glycolysis. Yet another prominent hit was the chloride (Cl^−^)/bicarbonate (HCO_3_^−^) anion exchanger SLC4A1 (band 3), whose overexpression decreased ATP specifically in glycolytic conditions. In RBCs, SLC4A1 associates with glycolytic enzymes under hypoxic conditions, and shunts glucose consumption from glycolysis to the PPP^54^. In summary, solute carriers have broad, albeit poorly understood, roles in regulating cellular ATP levels, and may have potential as therapeutic targets to restore energy.

Relatively, few hits increased ATP under basal conditions when both respiration and glycolysis are enabled, but these genes are of particular interest because they bypass feedback inhibition, and because they raise fascinating questions, such as the impact of supraphysiologic ATP levels. The most robust CRISPRa hit was *COL2A1*, a gene involved in collagen production with no known role in energy metabolism. Notable CRISRPi basal ATP hits included eukaryotic translation initiation factor 4 (EIF4G2), whose knockdown decreases mitochondrial gene expression in embryonic stem cells^55^. RAN, the highest CRISPRi basal hit, and KPNB1, a low ATP basal hit whose inhibition disrupts mitochondrial function and leads to cell death, both function in protein import to the nucleus^56^, demonstrating a potentially nuanced link between nuclear protein import and mitochondrial function. Overall, there were also relatively few low ATP basal hits. Interestingly, the two most prominent CRISPRi basal hits, *NMD3* (RNA export adaptor^57^) and the RNA-binding nuclear pore complex protein *NUP62* (ref. ^58^), increased ATP in respiratory conditions. Future studies will be required to determine how these genes maintain cellular ATP, and whether they play a role in regulating the relative contributions of ATP from respiration and glycolysis. As noted, there were no robust low ATP hits across all substrates in either CRISPRi or CRISPRa, indicating strong redundancy among pathways to maintain cellular ATP.

This work reveals genes and pathways that regulate energy levels, identifies whether these genes and pathways regulate energy through energy production or consumption, and informs the relative strength and metabolic context, in which these genes and pathways function. Our work shows that ATP investment in glycolysis and the PPP significantly contributes to ATP consumption, and may be responsible for eventual energy failure when downstream dysfunction prevents the energy debt from being repaid. Our work also shows on a systems level strong and reciprocal inhibitory interactions between respiration and glycolysis: knockdown of glycolysis-promoting enzymes strongly activates respiration, and this activation persists even in the absence of glycolytic ATP production. In particular, suppressing the HIF1 pathway resulted in the largest increase in respiration and, remarkably, we show that this pathway controls energy levels under non-hypoxic conditions, where it is believed to be inactive. Conversely, inhibition of respiratory chain function strongly bolsters glycolysis, even when only glycolytic substrates are present. As such, this work reveals potential directions to study energy control, including dissecting the molecular mechanisms underlying the respiration–glycolysis link, and the scope of HIF1 and other transcriptional pathways that regulate energy levels. Importantly, the impact of increased ATP on cellular growth, function, and survival depends heavily on the mechanism by which ATP is increased. Moreover, processes that disrupt energy metabolism frequently also disrupt other key metabolites such as NAD+ and reactive oxygen species, some of that could be studied using a similar approach.

Therapies that normalize energy metabolism have the potential to slow disease progression or prevent disease onset. However, major clinical trials targeting energy failure, such as those testing CoQ10, and creatine for Parkinson’s disease and Huntington’s disease, have failed^59–61^, while metabolic therapies, such as 2DG, metformin, and phenformin have had only modest success in cancer therapy^62,63^. Nonetheless, in rare instances bioenergetic therapies do provide major disease modifying, and even life-saving, benefit, including some of the same therapies that failed in other disease settings, for instance, CoQ10 therapy in individuals with defects in CoQ10 biosynthesis^64^, and a ketogenic diet to provide an alternative fuel source for patients with impaired glucose uptake into the brain (GLUT1 deficiency)^65^.

Similarly, metabolism-modulating genes represent tractable targets for cancer therapy^66^, although a systematic strategy for targeting metabolism in cancer has yet to be established. One promising therapy is the hexokinase substrate mannose^67^, and our data identify HK2 as a potent target to decrease ATP.

Our findings demonstrate that the efficacy of many metabolic therapies likely depends on the in vivo metabolic substrate composition, something that is usually poorly understood, and differs among cell types, energy requirements, stressors (e.g., starvation), and perhaps genetic modifiers of metabolism. In some cases, genetic therapies may be paired with specific metabolic therapies that enhance their efficacy. We hypothesize that genes that increase ATP by boosting energy production will enhance the function and survival in a substrate-specific manner, while some genes that boost ATP by decreasing energy consumption may increase survival at the expense of compromising energy-intensive cellular functions.

### Contact for reagent and resource sharing

Further information and requests for resources and reagents should be directed to Ken Nakamura (Ken.Nakamura@Gladstone.UCSF.edu).

## Methods

### Cell Lines

The ATP screen was conducted with K562 human female leukemia cells provided by Jonathan Weissman’s lab and identical to those used by Mendelsohn et al.^4^, with stable integration of dCas9-KRAB (CRISPRi) and dCas9-Suntag (CRISPRa). K562 cells were maintained at 37 °C in RPMI-1640 with 25 mM HEPES, 2.0 g/L NaHCO_3_, 0.3 g/L L-glutamine supplemented with 10% fetal bovine serum (FBS), 2 mM glutamine, 100 units/mL penicillin, and 100 mg/mL streptomycin.

Primary fibroblasts from patients with Leigh syndrome (PDSS2 male patient and NDUFS4 female patient) and a healthy control male patient were provided by Dr. Michio Hirano (Columbia) at early passage (p2–4). Fibroblasts were maintained at 37 °C in high-glucose DMEM/F12 with 4.5 g/L glucose, 0.11 g/L sodium pyruvate, 2.4 g/L NaHCO_3_, 0.3 g/L L-glutamine supplemented with 15% FBS, 100 units/mL penicillin, and 100 mg/mL streptomycin and passaged every 5–7 days. Primary fibroblasts were immortalized by lentivirus encoding neomycin-selectable hTERT. Fibroblasts plated at a density of 2000 cells/cm^2^ were transfected overnight in 2 µg/mL polybrene, and grown for 3 days prior to an additional 5 days selection in medium with 500 µg/mL geneticin. The surviving cells were expanded and frozen.

As stated in ref. ^4^, the human lung adenocarcinoma HCC827 cell line was originally obtained from Trever Bivona (UCSF) (ATCC 2868, 39-year-old Caucasian female individual). HCC827 cells were grown at 37 °C in RPMI medium with 10% FBS, 1% penicillin/streptomycin, and 1.5 mM pyruvate.

### Sequencing point mutations in Leigh syndrome lines

Genomic DNA from fibroblasts was purified with Qiagen’s DNeasy Blood and Tissue Kit (Cat. #69504). The regions spanning the mutation sites (exons 6 and 8 of *PDSS2*, and exons 2 and 3 of *NDUFS4*) were amplified with the following primers:

*PDSS2* exon 6: 5′-ACTGCACCTGGCCTGAAATA-3′/5′-CTTGTGCGAGAGTCCACAGA-3′

*PDSS2* exon 8: 5′-GCCTCAAGATCACTGGGAAA-3′/5′-CTTCTGGCGTGACAAGTGAA-3′

*NDUFS4* exon 2: 5′-CTACGTCCCCCTAAATAAACCAT-3′/5′-GTATCTTTGAGCACAGTGGTACT-3′

*NDUFS4* exon 3: 5′-ACAGAAAAAGGTATTCCAACTAACAGT-3′/5′-GGTAAACAGAGGTGTCAAAACTAG-3′

PCR products were purified with Qiagen’s QIAquick Gel Extraction Kit (Cat. #28704) and sequenced by Sanger method (Sequetech), then compared with the wild-type sequences (NM_020381.4 and NM_002495.4)

### ATP FACS screen and analysis

*Pooled CRISPRi/a perturbation*: Lentivirus (expressing the Clover-mApple ATP or dead sensors, or the CRISPRi sgRNA sublibrary) was produced by the UCSF Viracore and transduced with polybrene (8 µg/ml) into K562-dCas9-expressing cells via spinfection. Two days after transduction, puromycin selection (0.65 µg/ml) was maintained for 4–5 days total. Experiments were performed using the pooled cells surviving antibiotic section^4^.

*Flow cytometry*: To acutely force reliance on respiration and/or glycolysis prior to flow cytometry or luciferase experiments, cells were resuspended in PBS as follows^4^: (1) respiratory condition: 2% FBS, 10 mM pyruvate + 10 mM 2DG, (2) glycolytic condition: 2% FBS, 2 mM glucose + 5 µM oligo + 3 mM 2-deoxyglycose, or (3) basal condition: 2% FBS, 10 mM glucose + 5 mM pyruvate with no drugs. All flow cytometry experiments and screening were conducted on a BD FACSAria II. The donor fluorophore (Clover) was excited using a 488-nm laser and detected using a 525/50-nm filter. FRET was detected by excitation from the 488-nm laser and emission via a 610/20 or 615/30-nm filter. mTagBFP fluorescence from the CRISPRi library was excited by a 405-nm laser and detected by a 450/50-nm filter. Donor and FRET channels were detected on a linear scale, and mTagBFP fluorescence was detected on a logarithmic scale. Sorting was conducted using four-way purity into two tubes and an 85-µm nozzle. At least 200 cells/sgRNA in the sublibrary were collected in each repetition.

*DNA preparation and sequencing*: Cells collected by FACS were centrifuged, and pellets frozen at −20 °C until processing. Genomic DNA was isolated using the Macherey-Nagel NucleoBond Xtra Midi Plus (Macherey-Nagel, Germany). The sgRNAs were amplified and adaptors attached in a single PCR step. A total of 1.5 µg of undigested genomic DNA was used per 50 µL PCR reaction, and sufficient reactions were performed to include all isolated genomic DNA. PCR was conducted using Q5 HotStart High Fidelity Polymerase (NEB, Ipswich, MA) using forward primer: aatgatacggcgaccaccgaGATCGGAAGAGCACACGTCTGAACTCCAGTCACNNNNNNgcacaaaaggaaactcaccct and reverse primer: caagcagaagacggcatacgaCGACTCGGTGCCACTTTTTC, which include necessary adaptor and indexing sequences. *N* refers to the variable index sequence. PCR parameters were 98 °C for 30 s, followed by 26 cycles of 98 °C for 15 s, 62.5 °C for 15 s, 72 °C for 20 s, and ending with 72 °C for 6 min; samples were then ramped down to 4 °C and held. The resulting PCR product from multiple reactions were pooled, and unincorporated primers were removed using the GeneRead Size Selection Kit. Quality and purity of the PCR product was assessed by bioanalyzer (Agilent) and sequencing was performed on an Illumina HiSeq 2500 (ref. ^6^).

*Identification of hits*: A given gene perturbation’s ATP phenotype was the average enrichment of the strongest three sgRNA guides in the high versus low ATP fractions (log2 scale). Individual gene hits had ATP phenotypes more than three standard deviations from the mean ATP phenotype of quasi-genes. For each CRISPR sublibrary, quasi-genes phenotypes were calculated by randomly selecting and averaging nontargeting guides, generating a distribution of quasi-genes equal in number to the number of targeting transcripts. Individual sgRNAs that were two standard deviation hits with the dead sensor were considered artefacts and excluded from analysis. Genes were further assigned a Mann–Whitney *p*-value, where a lower *p*-value indicates greater concordance in phenotype between the ten sgRNAs targeting each gene. As such, a small *p*-value increased the confidence in the accuracy of a given ATP phenotype.

Genes were considered hits if they had ATP phenotypes with the ATP FRET sensor more than three standard deviations from the mean ATP phenotype of quasi-genes generated from randomly sampled nontargeting guides, and provided the sgRNAs did not alter the FRET signal of the dead sensor. Each gene was also assigned a *p*-value indicating the concordance in scores of the ten guides for each gene, with a *p*-value < 0.05 serving as a second measure of confidence in the robustness of hits (Supplementary Data 1). While using the *p*-value increased the specificity of hit identification from 99.3% to 99.9% for CRISPRa, and 99.6% to 99.9% for CRISPRi, it also substantially decreased the number of identified hits by ≈62% for CRISPRa, and 51% for CRISPRi.

*Gene function and pathway analyses*: Pre-ranked gene set enrichment analysis^68,69^ was used to determine enriched pathways and ontology terms among high and low ATP genes. The gene list was collapsed to unique gene identifiers, and were ranked based on the magnitude of their ATP phenotype. The maximum gene set size was set at 500 genes, and the minimum size at 10 genes. Thousand random sample permutations were carried out using the Molecular Signature Database c2 v6.2 and c5 v6.2, and a significance threshold was set at a nominal *p*-value of 0.05.

### Confirmation of gene knockdown

*qRT-PCR*: qRT-PCR gene relative expression quantifications were performed using 7900HT Fast Real-Time PCR System (Applied Biosystem) and using FAM-MGB TaqMan Gene Expression Assays (ThermoFisher, assay ID: Hs00606086_m1 – *HK2*, Hs00153153_m1—*HIF1A*, Hs01121918_m1—*ARNT*, Hs01060367_m1—*SENP1*, Hs01631624_gH—*VDAC1*, Hs00916521_m1—*SP1*, Hs00204417_m1—*NDUFA8*, and Hs00965587_m1—*SLC20A1*), together with VIC-MGB human *ACTB* (β-actin; ThermoFisher #4326315E) as endogenous control. cDNAs and PCR reactions were prepared according to the protocol for Cells-to-CT kit (ThermoFisher #AM1728), using the standard reverse transcription cycle (37 °C for 6 min, inactivation at 95 °C for 5 min, and hold at 4 °C), and qRT-PCR conditions (UDG incubation—50 °C for 2 min, enzyme activation—95 °C for 10 min, PCR cycle—95 °C for 15 s, and 60 °C for 1 min—repeat 40 cycles). All reactions were performed in a 384-well plate, in duplicate and from 2–3 independent experiment. CT (threshold cycle) values of each gene were averaged and calculated relatively to CT values of β-actin using the the 2^−ΔΔCT^ method.

*Western blot*: K562 cells were collected and lysed in RIPA buffer (ThermoFisher #89900) supplemented with Halt protease and phosphatase inhibitor cocktail (ThermoFisher #78444). Equal amounts of proteins (20 µg/lane) were separated by SDS–PAGE and transferred onto nitrocellulose membranes using iBlot™2 Dry Blotting System (ThermoFisher # IB23001 and #IB21001). Primary antibodies used recognized human HK1 antibody (ab150423, 1:2500, Abcam) and beta-actin antibody (MAB1501R, 1:5000, Millipore Sigma), followed by IRDye 800CW goat anti-Mouse IgG and IRDye 680RD goat anti-rabbit IgG secondary antibodies (Li-cor Biosciences, 926-32210 and 926-68071 1:10,000). Blots were visualized using the Odyssey infrared imaging system and analyzed using Image Studio Lite Software (Li-cor Biosciences).

### Bioenergetic function of CRISPRi/a hits

*Generation of individual Leigh and K562 lines expressing CRISPRi/a hits*: To enable CRISPRi effector domain-mediated transcriptional silencing in immortalized Leigh syndrome and control fibroblasts, we transduced cells with concentrated lentivirus expressing dCas9 fused to the repressive KRAB effector domain (dCas9-KRAB^70^) under a UCOE-EF1α promoter with a weak BFP fluorescent tag. Immortalized fibroblasts were plated at a high density of 6000 cells/cm^2^, and treated with 8 µg/mL polybrene after transduction. Two days after transfection, BFP-positive cells were isolated by FACS and expanded. After an additional passage, the cells were sorted again, and the high BFP-positive cells were isolated and expanded before freezing cell stocks. For both immortalized fibroblasts and K562 cells, one sgRNA/gene with the greatest phenotype in the ATP assay were chosen for selected CRISPRi high and low ATP hits, as well as control sgRNA. The sgRNAs were cloned by synthesizing two oligonucleotides with complementary sequences and overhangs per sgRNA. The oligonucleotides were annealed, and individually ligated into the sgRNA lentiviral backbone plasmid as in ref. ^6^, and sequences confirmed by Sanger sequencing of individual clones.

*Luciferase assays*: For Leigh fibroblast lines, 2000 cells were seeded per well in a 96-well plate. One day later, cells were treated with fresh culture media (baseline), or 2% FBS supplemented PBS along with 5 mM pyruvate + 10 mM glucose (basal), 10 mM pyruvate + 10 mM 2DG (respiratory), or 2 mM glucose + 5 µM oligo + 3 mM 2DG (glycolytic) for 1 h. For K562 cells, 20,000 cells were resuspended in either PBS (baseline) and flash frozen, or the above basal, respiratory, and glycolytic treatments for 1 h, before being flash frozen. Luciferase measurements of fibroblasts or thawed K562 cells were performed using the CellTiterGlo 2.0 kit and luminescence measured on a Spectramax M4 plate reader.

*Respiration and glycolysis*: The extracellular acidification rate (ECAR; a surrogate for glycolysis) and oxygen consumption rate (OCR; to assess mitochondrial respiration) were measured in immortalized fibroblast lines using a 96-well Seahorse XF96 Extracellular Flux Analyzer (Seahorse Bioscience). A Seahorse assay cartridge was calibrated with calibration medium overnight in a CO_2_-free incubator. Cells were seeded in XF96 microplates (10,000 cells/well) in DMEM complete medium supplemented with 15% FBS and 1 mM sodium pyruvate, and incubated overnight. Cells were washed and incubated with Seahorse XF base medium (supplemented with 25 mM glucose and 2 mM sodium pyruvate, pH = 7.4) and incubated in a CO_2_-free incubator for 1–2 h prior to assay. OCR and ECAR were measured at baseline and again after sequential addition of the respiratory inhibitors FCCP (1 µM, carbonyl cyanide-4-(trifluoromethoxy)phenylhydrazone, a protonophore that uncouples oxidative phosphorylation and then rotenone (1 µM, an inhibitor of complex I)). In parallel experiments, the ATP synthase inhibitor, oligomycin A (1 µM, an ATP synthase inhibitor) is added first, followed by rotenone. After each run, cells were fixed with 4% paraformaldehyde, and the OCR and ECAR signals were normalized to the number of cells in each well, estimated using DAPI staining. Maximal respiration was calculated by normalizing OCR values to the first basal OCR of the respective untransfected parent control line run in the same experiment.

### Functional characterization of a mini-library of ATP hits

*Generation of ATP hit mini-library*: Individual sgRNAs were selected to create a mini-library that could be screened more rapidly with analysis of fewer cells. From the primary screen, 1–3 sgRNAs/gene were selected based on robust decreased ATP phenotype (see Supplementary Data 3 for list of sgRNAs and genes). This mini-library included 246 sgRNAs targeting 150 genes and 18 nontargeting sgRNAs. sgRNAs were cloned by synthesizing two oligonucleotides with complementary sequences per sgRNA with necessary overhangs, annealing the oligonucleotides, and individually ligating into the sgRNA lentiviral backbone plasmid as used by Gilbert et al.^6^. Sequences were confirmed by Sanger sequencing of individual clones. DNA from individual sgRNAs was then pooled in approximately equal amounts. Lentivirus was created and cells were selected for integration of the sgRNA-bearing sequence as in the primary screen.

*Mitochondrial content*: K562 cells expressing dCas9-KRAB and either individual sgRNA of interest or the mini-library of ATP hits were preincubated in basal conditions, as in the primary screen. After preincubation, cells were stained with MitoTracker Green FM at 20 nM for 30 min before washing and resuspending in basal conditions.

To screen for ATP-impacting genes that act primarily by regulating mitochondrial content, the top and bottom 25% of K562 cells expressing the mini-library of ATP hits were sorted based on mitochondrial content corrected for cell size (FITC/FSC-A). Genomic DNA from each cell fraction was isolated, sequenced, and sgRNA read counts quantified. The impact of each sgRNA on mitochondrial content was determined by first normalizing the number of counts for a given sgRNA to the total counts of nontargeting guides (performed on a per-sample basis), and calculating the fold change in normalized counts between high- and low-MitoTracker fractions, yielding a mitochondrial content phenotype. In order to quantify mitochondrial content in individual lines, FITC/FSC-A was measured after basal condition pretreatment, as in the primary screen.

*ATP consumption*: K562 cells expressing dCas9-KRAB, the ATP sensor, and either individual sgRNA of interest or the mini-library of ATP hits were preincubated in basal, respiratory, or glycolytic conditions, as in the primary screen. After preincubation, drugs were added to block ATP production (5 µM oligo, 10 mM 2DG, and 1 mM IA). In order to screen for ATP-impacting genes that act primarily by regulating ATP consumption, the top and bottom quartiles of K562 cells expressing the mini-library of ATP hits were sorted based on ATP level 2 min after blocking all ATP production. Genomic DNA from each cell fraction was isolated, sequenced, and sgRNA read counts quantified. The impact of each sgRNA on ATP during ATP depletion was determined by first normalizing the number of counts for a given sgRNA to the total counts of nontargeting guides within a sample, and calculating the fold change in normalized counts between high and low ATP fractions, yielding an ATP phenotype similar to that calculated for the primary screen. The ATP consumption phenotype was calculated as the ratio between the ATP phenotype post-ATP depletion versus pre-ATP depletion. Hit genes are those with depletion phenotypes more than three standard deviations from that of the nontargeting sgRNA included in the mini-library. Hits with more enrichment in the high ATP fraction post-ATP depletion indicate slower ATP consumption, hits enriched in the low ATP fraction post-ATP depletion indicate faster ATP consumption.

In order to quantify ATP consumption in individual lines, mean ATP level was measured by FACS before and after blocking ATP production every 2 min, for up to 8–16 min. For a low ATP consumption control, cells were incubated for 24 h in 10 µM cycloheximide. ATP consumption rate was compared between lines and a no-knockdown control line by linear regression *F*-test.

### Metabolomics by LC–MS

K562 cells were incubated for 2 and 6 h in either (1) respiratory condition: 2% FBS, 10 mM [U-^13^C]pyruvate + 10 mM 2DG, (2) glycolytic condition: 2% FBS, 2 mM [U-^13^C]glucose + 5 µM oligomycin + 3 mM 2-deoxyglycose, (3) basal condition: 2% FBS, 10 mM [U-^13^C]glucose + 5 mM pyruvate with no drugs, or (4) 2% FBS, 10 mM [U-^13^C]pyruvate alone, before metabolite extraction in 80% methanol and drying in a Labconco CentriVap. Dried metabolites were resuspended in 50% ACN:water and loaded onto a Luna 3um NH2 100 A (150 × 2.0 mm) column (Phenomenex). The chromatographic separation was performed on a Vanquish Flex (Thermo Scientific) with mobile phases A (5 mM NH4AcO, pH 9.9) and B (ACN) and a flow rate of 200 μL/min. A linear gradient from 15% A to 95% A over 18 min was followed by 9 min isocratic flow at 95% A and reequilibration to 15% A. Metabolites were detection with a Q Exactive mass spectrometer (Thermo Scientific) run with polarity switching (+3.5 kV/−3.5 kV) in full scan mode with an *m*/*z* range of 65–975. TraceFinder 4.1 (Thermo Scientific) was used to quantify the targeted metabolites by area under the curve, using expected retention time and accurate mass measurements (<5 p.p.m.). Values were normalized to cell number. Relative amounts of metabolites were calculated by summing up the values for all isotopologues of a given metabolite. Metabolite isotopologue distributions were corrected for natural C13 abundance.

*Growth screen and ATP*: We generated HCC827 human lung cancer cells expressing dCas9-KRAB, and transduced them with the mini-library of ATP hits. After lentiviral transduction followed by puromycin selection for 5 days, HCC827 cells were grown in basal (standard media as described above), glycolytic (oligo 5 µM), and respiratory (2DG 10 mM) metabolic conditions. After 3 days of growth under these conditions, cells were collected and genomic DNA from each plate was isolated, sequenced, and sgRNA read counts quantified. The impact of each sgRNA on growth was determined by first normalizing the number of counts for a given sgRNA to the total counts of nontargeting guides within a sample, and calculating the fold change in normalized counts post-growth versus pre-growth.

Growth phenotypes were compared to ATP phenotypes in HCC827 cells, determined by performing the ATP FACS screen in cells transduced with the mini-library of ATP hits. ATP phenotypes were determined by normalizing the number of counts for each sgRNA to the total number of nontargeting guides within a sample, and calculating the fold change in normalized counts in high ATP versus low ATP cell fractions.

### Quantification and statistical analysis

All statistical analyses, including the *n*, what *n* represents, description of error bars, statistical tests used, and level of significance, are stated in the figure legends. All measurements were taken from distinct samples. In figure legends, “replicates” denotes biological replicates. FACS analyses were graphed with box plots, with the top and bottom of the box indicating the interquartile range, the whiskers the 5th and 95th percentiles, and the center line in the box the median. One- or two-way ANOVA and multiple comparisons tests were used to compare ATP levels, assessed with either luciferase or the ATP FRET sensor-based assays, with a *p* < 0.05 considered significant. Correlation analyses were performed with linear regression after removal of outliers using the ROUT method^71^, and determined to have nonzero slopes via *F*-test. For the respiratory condition, hits were defined as genes with two or more sgRNAs >3 SD beyond the phenotype of the nontargeting sgRNAs after averaging the three repetitions of the respiratory condition screen. We excluded hits that showed a phenotype by these same criteria in the same direction with the dead mutant sensor. For the glycolytic and basal conditions, which had narrower distributions of the nontargeting sgRNAs, we defined a hit as having three or more sgRNAs >3 SD beyond the average phenotype of the nontargeting sgRNAs, and removed sgRNAs >2 SD beyond nontargeting sgRNAs with the dead sensor. Hits can be further filtered by those with high agreement between targeting sgRNA (Mann–Whitney *p*-value < 0.05, bolded in Supplementary Data 1), which marginally increases the screen specificity, calculated using the number of quasi-gene hits to estimate the false positive rate, but substantially decreases the number of detected hits. Phenotypes (mean of best three sgRNAs averaged over all repetitions) for all screened genes in all conditions are provided in Supplementary Data 1. GraphPad Prism and FlowJo were used to generate graphs, and GraphPad Prism was used for statistical analyses.

The ATP phenotype *Z*-score was calculated based on the top three sgRNA that did not have significant phenotypes with the inactive ATP sensor. ATP *Z*-scores were calculated based on the difference between a given gene’s phenotype and the distribution of the nontargeting guides included in the gene’s CRISPR sublibrary.

### Reporting summary

Further information on research design is available in the Nature Research Reporting Summary linked to this article.

## Supplementary information

Supplementary Information

Peer Review File

Supplementary Data 1

Supplementary Data 2

Supplementary Data 3

Supplementary Data 4

Reporting Summary

Description of Additional Supplementary Files

## Data Availability

All relevant data are available from the authors, and source data are provided as a Source data file (10.6084/m9.figshare.12678938).

## Source data

Source Data

## Code availability

Software for processing and analysis of sequencing reads from the pooled library was the same as that used by Gilbert et al.^6^. For the ATP FRET-based screen, phenotypes (average of strongest sgRNAs) for all genes screened in all conditions are provided in Supplementary Data 1, 3, and 4. Sequences for all sgRNAs were published by Gilbert et al.^6^.

## References

[1] Pathak D, Berthet A, Nakamura K (2013). Energy failure: does it contribute to neurodegeneration?. Ann. Neurol..

[2] Ganapathy-Kanniappan S, Geschwind JF (2013). Tumor glycolysis as a target for cancer therapy: progress and prospects. Mol. Cancer.

[3] Koopman WJ, Willems PH, Smeitink JA (2012). Monogenic mitochondrial disorders. N. Engl. J. Med..

[4] Mendelsohn BA (2018). A high-throughput screen of real-time ATP levels in individual cells reveals mechanisms of energy failure. PLoS Biol..

[5] Vander Heiden MG (2010). Evidence for an alternative glycolytic pathway in rapidly proliferating cells. Science.

[6] Gilbert LA (2014). Genome-scale CRISPR-mediated control of gene repression and activation. Cell.

[7] Danhauser K (2015). Treatment options for lactic acidosis and metabolic crisis in children with mitochondrial disease. J. Inherit. Metab. Dis..

[8] Chatfield KC (2015). Mitochondrial energy failure in HSD10 disease is due to defective mtDNA transcript processing. Mitochondrion.

[9] Zschocke J (2012). HSD10 disease: clinical consequences of mutations in the HSD17B10 gene. J. Inherit. Metab. Dis..

[10] Lustbader JW (2004). ABAD directly links Abeta to mitochondrial toxicity in Alzheimer’s disease. Science.

[11] Horlbeck MA (2018). Mapping the genetic landscape of human cells. Cell.

[12] Yang J (2012). Human CHCHD4 mitochondrial proteins regulate cellular oxygen consumption rate and metabolism and provide a critical role in hypoxia signaling and tumor progression. J. Clin. Invest..

[13] Ryu J-H (2011). Hypoxia-inducible factor α subunit stabilization by NEDD8 conjugation is reactive oxygen species-dependent. J. Biol. Chem..

[14] Zhang D, Yip YM, Li L (2016). In silico construction of HK2-VDAC1 complex and investigating the HK2 binding-induced molecular gating mechanism of VDAC1. Mitochondrion.

[15] Beishline K, Azizkhan-Clifford J (2015). Sp1 and the ‘hallmarks of cancer’. FEBS J..

[16] Mueller CL, Jaehning JA (2002). Ctr9, Rtf1, and Leo1 are components of the Paf1/RNA polymerase II complex. Mol. Cell Biol..

[17] Yu JS, Cui W (2016). Proliferation, survival and metabolism: the role of PI3K/AKT/mTOR signalling in pluripotency and cell fate determination. Development.

[18] Cornejo, M. P. et al. Neuroendocrine regulation of metabolism. *J. Neuroendocrinol.* **28**, 1–12 (2016). 10.1111/jne.12395 PMC4956544 27114114

[19] Quan XJ, Liang CL, Sun MZ, Zhang L, Li XL (2019). Overexpression of steroid receptor coactivators alleviates hyperglycemia-induced endothelial cell injury in rats through activating the PI3K/Akt pathway. Acta Pharm. Sin..

[20] Sugita A (2011). Cellular ATP synthesis mediated by type III sodium-dependent phosphate transporter Pit-1 is critical to chondrogenesis. J. Biol. Chem..

[21] Deng H, Huang X, Yuan L (2016). Molecular genetics of the COL2A1-related disorders. Mutat. Res. Rev. Mutat. Res..

[22] Brahimi-Horn MC (2012). Expression of a truncated active form of VDAC1 in lung cancer associates with hypoxic cell survival and correlates with progression to chemotherapy resistance. Cancer Res..

[23] Minet E (1999). HIF1A gene transcription is dependent on a core promoter sequence encompassing activating and inhibiting sequences located upstream from the transcription initiation site and cis elements located within the 5’UTR. Biochem. Biophys. Res. Commun..

[24] Lopez LC (2006). Leigh syndrome with nephropathy and CoQ10 deficiency due to decaprenyl diphosphate synthase subunit 2 (PDSS2) mutations. Am. J. Hum. Genet..

[25] Miettinen TP, Bjorklund M (2016). Cellular allometry of mitochondrial functionality establishes the optimal cell size. Dev. Cell.

[26] Geisler S (2010). PINK1/Parkin-mediated mitophagy is dependent on VDAC1 and p62/SQSTM1. Nat. Cell Biol..

[27] Depaoli MR (2018). Real-time imaging of mitochondrial ATP dynamics reveals the metabolic setting of single cells. Cell Rep..

[28] DeWaal D (2018). Hexokinase-2 depletion inhibits glycolysis and induces oxidative phosphorylation in hepatocellular carcinoma and sensitizes to metformin. Nat. Commun..

[29] Fan J (2014). Quantitative flux analysis reveals folate-dependent NADPH production. Nature.

[30] Sullivan LB (2015). Supporting aspartate biosynthesis is an essential function of respiration in proliferating cells. Cell.

[31] Muz B, de la Puente P, Azab F, Azab AK (2015). The role of hypoxia in cancer progression, angiogenesis, metastasis, and resistance to therapy. Hypoxia (Auckl.).

[32] Papandreou I, Cairns RA, Fontana L, Lim AL, Denko NC (2006). HIF-1 mediates adaptation to hypoxia by actively downregulating mitochondrial oxygen consumption. Cell Metab..

[33] Brand MD, Nicholls DG (2011). Assessing mitochondrial dysfunction in cells. Biochem J..

[34] Atkinson DE, Walton GM (1967). Adenosine triphosphate conservation in metabolic regulation. Rat liver citrate cleavage enzyme. J. Biol. Chem..

[35] Jain IH (2016). Hypoxia as a therapy for mitochondrial disease. Science.

[36] Okamoto A (2017). HIF-1-mediated suppression of mitochondria electron transport chain function confers resistance to lidocaine-induced cell death. Sci. Rep..

[37] Buttgereit F, Brand MD (1995). A hierarchy of ATP-consuming processes in mammalian cells. Biochem. J..

[38] Lin R (2015). 6-Phosphogluconate dehydrogenase links oxidative PPP, lipogenesis and tumour growth by inhibiting LKB1-AMPK signalling. Nat. Cell Biol..

[39] Patra KC (2013). Hexokinase 2 is required for tumor initiation and maintenance and its systemic deletion is therapeutic in mouse models of cancer. Cancer Cell.

[40] Tanner LB (2018). Four key steps control glycolytic flux in mammalian cells. Cell Syst..

[41] Sun Y (2019). Functional genomics reveals synthetic lethality between phosphogluconate dehydrogenase and oxidative phosphorylation. Cell Rep..

[42] Wilson JE (2003). Isozymes of mammalian hexokinase: structure, subcellular localization and metabolic function. J. Exp. Biol..

[43] Colell A (2007). GAPDH and autophagy preserve survival after apoptotic cytochrome c release in the absence of caspase activation. Cell.

[44] McIver, S. C. et al. Exosome complex orchestrates developmental signaling to balance proliferation and differentiation during erythropoiesis. *Elife* **5**, e17877 (2016). 10.7554/eLife.17877 PMC5040589 27543448

[45] Rehage N (2018). Binding of NUFIP2 to Roquin promotes recognition and regulation of ICOS mRNA. Nat. Commun..

[46] Dermody JL, Buratowski S (2010). Leo1 subunit of the yeast paf1 complex binds RNA and contributes to complex recruitment. J. Biol. Chem..

[47] Guillaumond F (2010). Kruppel-like factor KLF10 is a link between the circadian clock and metabolism in liver. Mol. Cell Biol..

[48] Zhang L (2015). KLF15 establishes the landscape of diurnal expression in the heart. Cell Rep..

[49] Zhou X, Paredes JA, Krishnan S, Curbo S, Karlsson A (2015). The mitochondrial carrier SLC25A10 regulates cancer cell growth. Oncotarget.

[50] Scalise M, Pochini L, Galluccio M, Indiveri C (2016). Glutamine transport. From energy supply to sensing and beyond. Biochim. Biophys. Acta.

[51] Rubio-Aliaga I, Wagner CA (2016). Regulation and function of the SLC38A3/SNAT3 glutamine transporter. Channels (Austin).

[52] Liu Y (2012). A small-molecule inhibitor of glucose transporter 1 downregulates glycolysis, induces cell-cycle arrest, and inhibits cancer cell growth in vitro and in vivo. Mol. Cancer Ther..

[53] Pullen TJ (2012). Overexpression of monocarboxylate transporter-1 (SLC16A1) in mouse pancreatic beta-cells leads to relative hyperinsulinism during exercise. Diabetes.

[54] Chu H (2016). Reversible binding of hemoglobin to band 3 constitutes the molecular switch that mediates O2 regulation of erythrocyte properties. Blood.

[55] Yoffe Y (2016). Cap-independent translation by DAP5 controls cell fate decisions in human embryonic stem cells. Genes Dev..

[56] Zhu ZC, Liu JW, Li K, Zheng J, Xiong ZQ (2018). KPNB1 inhibition disrupts proteostasis and triggers unfolded protein response-mediated apoptosis in glioblastoma cells. Oncogene.

[57] Buhlmann M (2015). NMD3 regulates both mRNA and rRNA nuclear export in African trypanosomes via an XPOI-linked pathway. Nucleic Acids Res..

[58] Dargemont C, Schmidt-Zachmann MS, Kuhn LC (1995). Direct interaction of nucleoporin p62 with mRNA during its export from the nucleus. J. Cell Sci..

[59] Beal MF (2014). A randomized clinical trial of high-dosage coenzyme Q10 in early Parkinson disease: no evidence of benefit. JAMA Neurol..

[60] McGarry A (2017). A randomized, double-blind, placebo-controlled trial of coenzyme Q10 in Huntington disease. Neurology.

[61] Writing Group for the NETiPDN-PI, et al. Effect of creatine monohydrate on clinical progression in patients with Parkinson disease: a randomized clinical trial. *JAMA* **313**, 584–593 (2015). 10.1001/jama.2015.120 PMC4349346 25668262

[62] Raez LE (2013). A phase I dose-escalation trial of 2-deoxy-D-glucose alone or combined with docetaxel in patients with advanced solid tumors. Cancer Chemother. Pharm..

[63] Zi F (2018). Metformin and cancer: an existing drug for cancer prevention and therapy. Oncol. Lett..

[64] Hirano M, Garone C, Quinzii CM (2012). CoQ(10) deficiencies and MNGIE: two treatable mitochondrial disorders. Biochim. Biophys. Acta.

[65] Veyrat-Durebex C (2018). How can a ketogenic diet improve motor function?. Front. Mol. Neurosci..

[66] Sharma H (2018). Development of novel therapeutics targeting isocitrate dehydrogenase mutations in cancer. Curr. Top. Med. Chem..

[67] Gonzalez PS (2018). Mannose impairs tumour growth and enhances chemotherapy. Nature.

[68] Subramanian A (2005). Gene set enrichment analysis: a knowledge-based approach for interpreting genome-wide expression profiles. Proc. Natl Acad. Sci. USA.

[69] Mootha VK (2003). PGC-1alpha-responsive genes involved in oxidative phosphorylation are coordinately downregulated in human diabetes. Nat. Genet..

[70] Gilbert LA (2013). CRISPR-mediated modular RNA-guided regulation of transcription in eukaryotes. Cell.

[71] Motulsky HJ, Brown RE (2006). Detecting outliers when fitting data with nonlinear regression-a new method based on robust nonlinear regression and the false discovery rate. BMC Bioinformatics.

