# Supplementary material for: Defining the ATPome reveals cross-optimization of metabolic pathways
Source: Nat Commun. 2020 Aug 28;11:4319. doi: 10.1038/s41467-020-18084-6 (PMC7455733; doi:10.1038/s41467-020-18084-6)
Supplement: Supplementary file 1 — Supplementary Information [file 41467_2020_18084_MOESM1_ESM.pdf]

Defining the ATPome Reveals Cross-Optimization of Metabolic Pathways

Bennett et al.

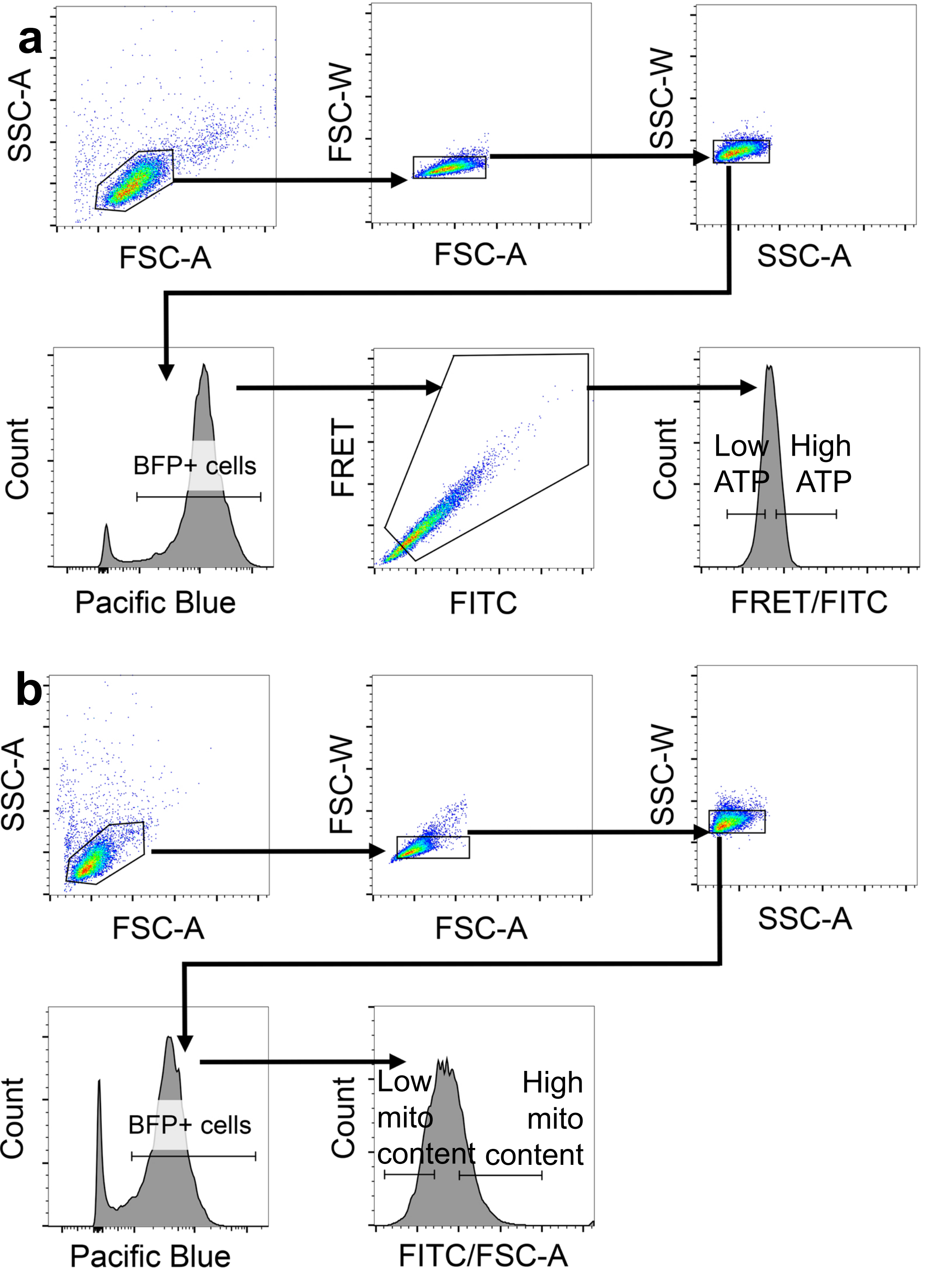

a
SSC-A
FSC-A
FSC-W
FSC-A
SSC-W
SSC-A
Count
BFP+ cells
Pacific Blue
FRET
FITC
Count
Low ATP
High ATP
FRET/FITC
b
SSC-A
FSC-A
FSC-W
FSC-A
SSC-W
SSC-A
Count
BFP+ cells
Pacific Blue
Count
Low mito content
High mito content
FITC/FSC-A

Figure S1. Gating strategies used for cell sorting.

(a) Cells were first gated by forward (FSC-A) and side scatter (SSC-A), and then for single cells using FSC-A/FSC-W and SSC-A/SSC-W. Next, cells were gated based on positive BFP fluorescence, indicating the presence of a CRISPRi or CRISPRa sgRNA, followed by gating out of cells with donor or FRET signal from the ATP sensor that overlapped with background (too dim) or exceeded the range of the sorter (too bright). The ratio of FRET/Donor was displayed as a histogram, and the top and bottom 25% of cells on this histogram were separated by FACS and collected for sequencing, or measurement of ATP level.

(b) A similar gating strategy is used for measuring mitochondrial content. After gating on positive BFP fluorescence, the ratio of FITC/FSC-A was displayed as a histogram, and the top and bottom 25% of cells were separated by FACS and collected for sequencing.

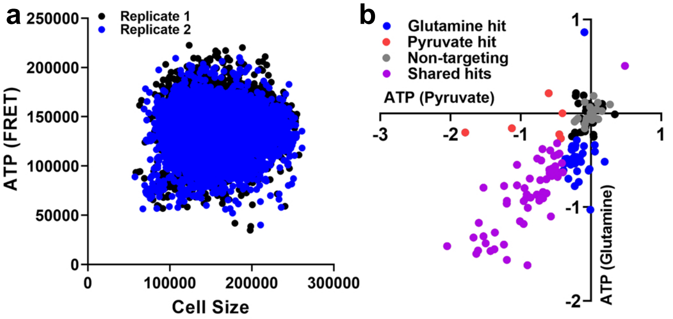
a
Replicate 1
Replicate 2
250000
200000
150000
100000
50000
0
ATP (FRET)
0
100000
200000
300000
Cell Size
b
Glutamine hit
Pyruvate hit
Non-targeting
Shared hits
ATP (Pyruvate)
-3
-2
-1
1
1
-1
-2
ATP (Glutamine)

Figure S2. ATP phenotypes are highly correlated across respiratory substrates but independent of cell size.

(a) K562 cells stably expressing dCas9-KRAB (CRISPRi) and the ATP FRET sensor were sorted under basal conditions. There was no correlation between cell size and ATP FRET signal. Plotted are two replicates of sorted sublibraries (replicate 1 Pearson $r = 0.007$, $n = 7452$ $p = 0.515$; replicate 2 $r = 0.020$, $n = 7449$, $p = 0.0787$ for non-zero slope or null hypothesis of no correlation by Pearson's correlation test).

(b) Individual sgRNA ATP phenotypes averaged from two replicates of a mini-library of 184 sgRNA (76 genes, 19 non-targeting sgRNA) in respiratory conditions where the metabolic substrate was either 10mM pyruvate or 2mM glutamine. 60/67 sgRNA with hit phenotypes with pyruvate were also hits with glutamine (greater than 3 standard deviations from the non-targeting guide average). 2 replicates per group, with 1M cells/sample. Source data, exact p-values, and 95% confidence intervals are provided as a Source Data file.

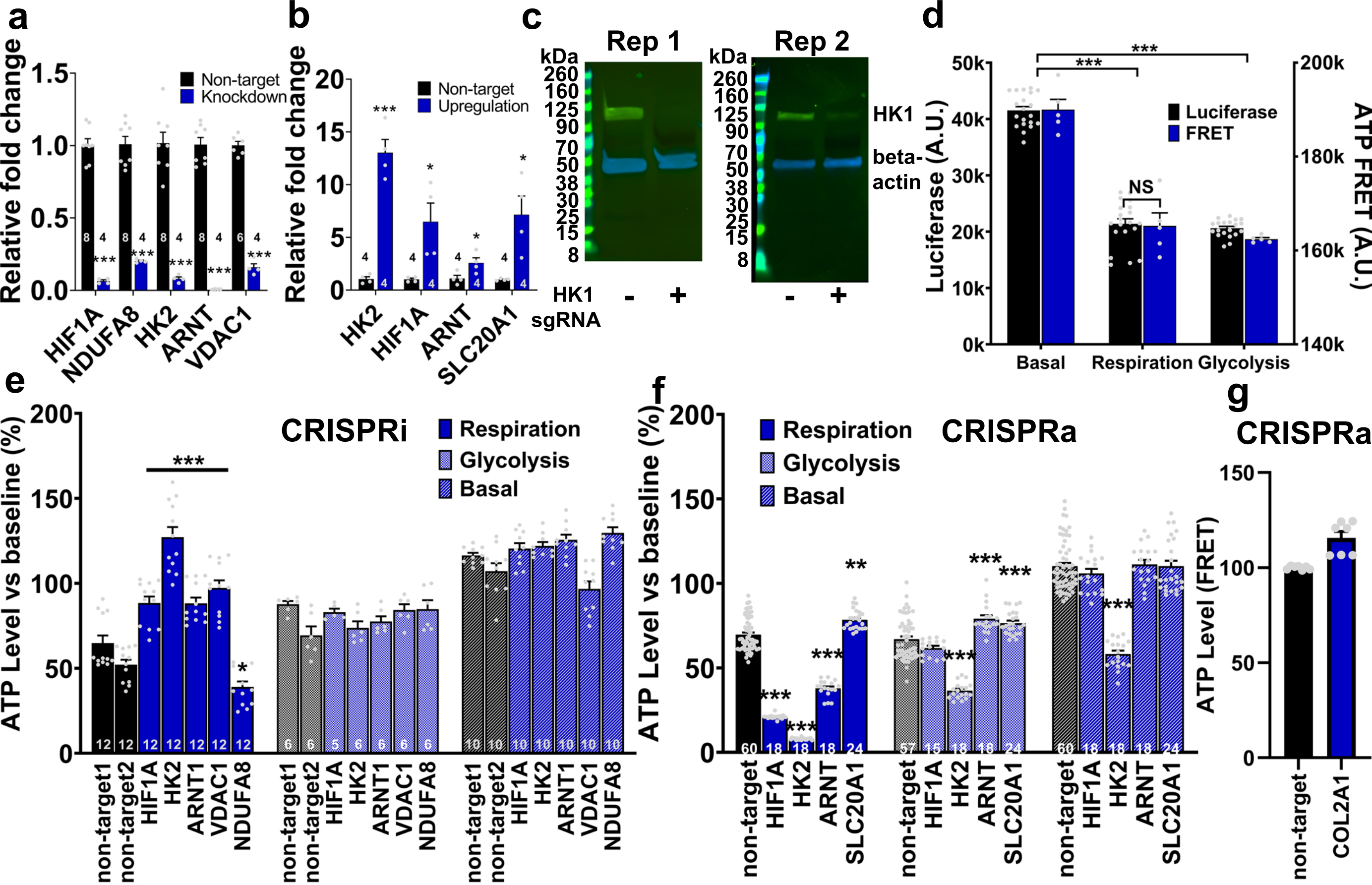
a
Relative fold change
Non-target
Knockdown
HIF1A
NDUFA8
HK2
ARNT
VDAC1
b
Relative fold change
Non-target
Upregulation
HK2
HIF1A
ARNT
SLC20A1
c
Rep 1
Rep 2
kDa
HK1
beta-actin
HK1 sgRNA
d
Luciferase (A.U.)
ATP FRET (A.U.)
Luciferase
FRET
NS
Basal
Respiration
Glycolysis
e
CRISPRi
Respiration
Glycolysis
Basal
ATP Level vs baseline (%)
non-target1
non-target2
HIF1A
HK2
ARNT1
VDAC1
NDUFA8
f
CRISPRa
ATP Level vs baseline (%)
Respiration
Glycolysis
Basal
non-target
HIF1A
HK2
ARNT
SLC20A1
g
CRISPRa
ATP Level (FRET)
non-target
COL2A1

Figure S3. Validation of ATP hits (CRISPRi and CRISPRa), Related to Figure 3.

(a,b) K562 cells expressing dCas9-KRAB (CRISPRi) or dCas9-Suntag (CRISPRa) were stably transduced with a single sgRNA targeting the indicated gene. Data show relative fold-changes in gene transcription versus β-actin analyzed by RT-qPCR (quantitative real-time reverse transcription PCR) for (a) CRISPRi or (b) CRISPRa. All data were analyzed from the total number of technical replicates shown in the bars, in at least two experiments per line. *$p < 0.05$, ***$p < 0.001$ versus non-targeting control, multiple two-sided t-tests with Holm-Sidak correction for multiple comparisons.

(c) CRISPRi sgRNA targeting HK1 in K562 cells robustly decrease HK1 expression, measured by western blot in n=2 replicates. The samples derive from the same experiment and the gels were processed in parallel.

(d) ATP measured in K562 cells both by flow cytometry with the ATP FRET sensor and by luciferase assay. ATP levels were assessed after 1 hr pre-incubation with basal, respiration-only (10 mM 2DG, and 10 mM pyruvate) or glycolysis-only (5 μM oligo) substrates, and the changes expressed relative to pre-treatment levels. ***$p < 0.001$ versus both control and blocked glycolysis/respiration groups for both ATP measuring methods by two-way ANOVA with Tukey multiple comparisons test; n = 5 samples of 10,000 cells/group for FACS in three experiments; n = 20 replicates of 20,000 cells/group for luciferase in two experiments.

(e,f) K562 cells expressing individual ATP hits targeted by CRISPRi (e) and CRISPRa (f) were acutely incubated in basal, respiratory or glycolytic conditions. Bars represent ATP levels, measured by luciferase, in drug/substrate treatment (respiratory or glycolytic) relative to controls. **$p < 0.01$, ***$p < 0.001$ by two-way ANOVA with Dunnett's multiple comparisons test. The data were analyzed from the total replicates shown in the bars in three experiments.

(g) ATP measured by FRET in K562 cells overexpressing *COL2A1* by CRISPRa incubated in basal conditions. ***$p < 0.001$ versus non-targeting control by unpaired two-sided Student's t-test; n = 7 replicates of 10,000 cells/group for FACS in two experiments. Data are presented as

mean values ± SEM. Source data, exact p-values and 95% confidence intervals are provided as a Source Data file.

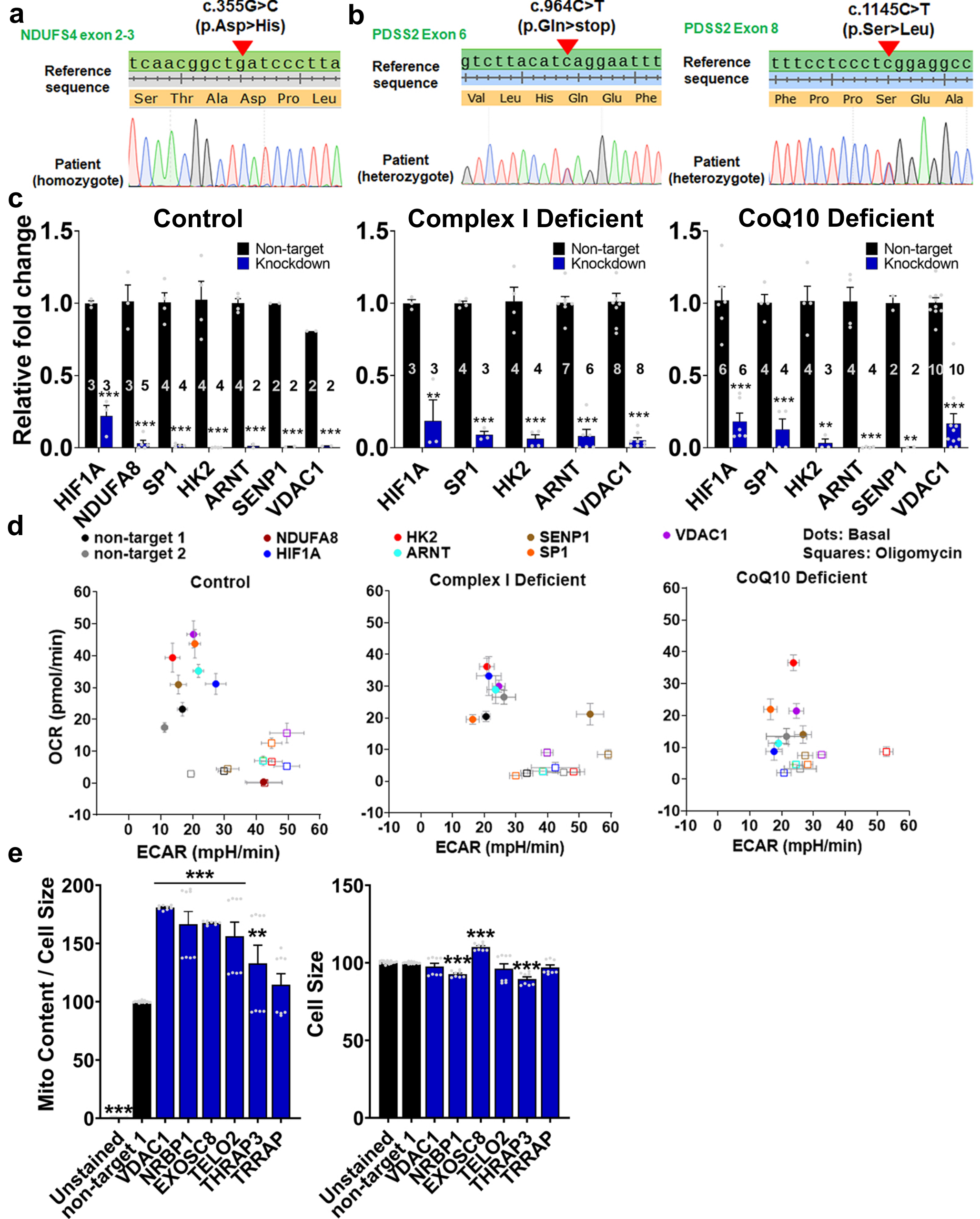
a
NDUFS4 exon 2-3
c.355G>C
(p.Asp>His)
Reference sequence
tcaacggctgatcccttta
Ser Thr Ala Asp Pro Leu
Patient (homozygote)
b
PDSS2 Exon 6
c.964C>T
(p.Gln>stop)
Reference sequence
gtcttacatcaggaattt
Val Leu His Gln Glu Phe
Patient (heterozygote)
PDSS2 Exon 8
c.1145C>T
(p.Ser>Leu)
Reference sequence
tttcctccctcggaggcc
Phe Pro Pro Ser Glu Ala
Patient (heterozygote)
c
Control
Complex I Deficient
CoQ10 Deficient
Relative fold change
Non-target
Knockdown
HIF1A
NDUFA8
SP1
HK2
ARNT
SENP1
VDAC1
d
non-target 1
non-target 2
NDUFA8
HIF1A
HK2
ARNT
SENP1
SP1
VDAC1
Dots: Basal
Squares: Oligomycin
Control
Complex I Deficient
CoQ10 Deficient
OCR (pmol/min)
ECAR (mph/min)
e
Mito Content / Cell Size
Cell Size
Unstained
non-target 1
VDAC1
NRBP1
EXOSC8
TELO2
THRAP3
TRRAP

Figure S4. Validation of high ATP respiratory hits in K562 and respiratory deficient cells, Related to Figure 4.

(a-b) Chromatogram results of Sanger sequencing show one homozygous point mutation in the *NDUFS4* patient fibroblast line(c.355C>G), and the two heterozygous point mutations in the *PDSS2* patient fibroblast cell line (c.964C>T and c.1145C>T). Arrows represent the positions of the mutations.

(c) Relative fold change in transcription of knockdown lines in control and patient fibroblasts, analyzed by RT-qPCR. Data represent the fold change in the knockdown gene's transcript relative to β-actin transcript level, calculated using the $2^{-\Delta\Delta Ct}$ method. All data were analyzed from the total number of technical replicates shown in the bars, in at least two experiments per line. $^{**}p < 0.01$, $^{***}p < 0.001$ versus non-targeting control, multiple two-sided t-tests with Holm-Sidak correction for multiple comparisons.

(d) Space plot of mitochondrial respiration rate (oxygen consumption rate, OCR) and glycolytic rate (extracellular acidification rate, ECAR) of knockdown lines in control and patient fibroblasts, under basal conditions, and following inhibition of respiration (oligomycin). Most CoQ10 deficient cells have decreased basal respiration and increased glycolysis. Number of replicates for each condition is noted in the Source Data file.

(e) Validation of changes in mitochondrial content in K562 cells. Mitochondrial content (FITC, MitoTracker Green) and cell size (FSC-A) were quantified in K562 cells expressing individual sgRNAs in basal substrate. Unstained and non-targeting n = 16, targeting line n = 8 replicates. $^{*}p < 0.05$, $^{**}p < 0.01$, $^{***}p < 0.001$ by one-way ANOVA with Dunnett multiple comparisons test. Data are presented as mean values ± SEM. Source data and exact p-values are provided as a Source Data file.

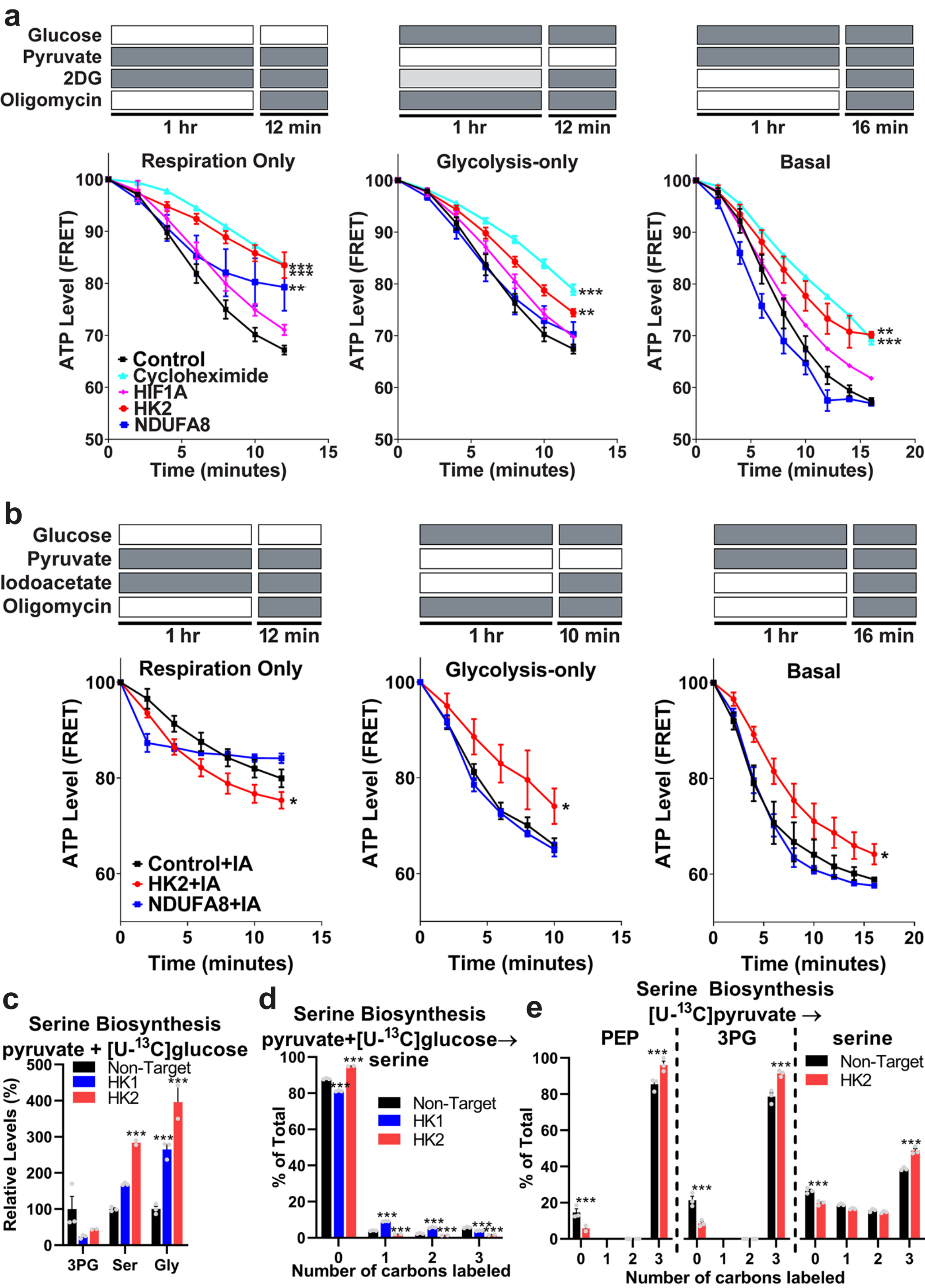

a
Glucose
Pyruvate
2DG
Oligomycin
1 hr
12 min
16 min
Respiration Only
Glycolysis-only
Basal
ATP Level (FRET)
Time (minutes)
Control
Cycloheximide
HIF1A
HK2
NDUFA8
b
Glucose
Pyruvate
Iodoacetate
Oligomycin
1 hr
12 min
10 min
16 min
Respiration Only
Glycolysis-only
Basal
Control+IA
HK2+IA
NDUFA8+IA
c
Serine Biosynthesis
pyruvate + [U-13C]glucose
Relative Levels (%)
Non-Target
HK1
HK2
3PG
Ser
Gly
d
Serine Biosynthesis
pyruvate+[U-13C]glucose→ serine
% of Total
Number of carbons labeled
e
Serine Biosynthesis
[U-13C]pyruvate →
PEP
3PG
serine
Number of carbons labeled

Figure S5. HK2 differentially impacts ATP consumption and serine biosynthesis depending on metabolic substrate, related to Figures 4d and 5.

(a) HK2 increases ATP Consumption by metabolizing 2DG. K562 cell lines expressing individual sgRNAs were pre-incubated in respiratory, glycolytic or basal conditions. The rate of ATP consumption was then estimated as the rate of decline in ATP FRET signal when all ATP production was blocked with 10 mM 2DG and 5 µM oligo. Inhibiting protein synthesis with cycloheximide (10 µg/mL 24h, CHX) and knocking down HK2 decreased consumption in all metabolic conditions. Control n = 6, HIF1A n = 4, HK2 n = 5, NDUFA8 n = 4, CHX n = 4 replicates of 10,000 cells per timepoint. **p < 0.01, ***p < 0.001 by two-sided F-test for linear regression versus control.

(b) ATP consumption was estimated after blocking all ATP production with 1mM iodoacetate (IA) and 5uM oligo (without 2DG). HK2 knockdown still decreases ATP consumption when glucose is present, but it increases consumption in respiratory conditions that lack glucose. n = 4 biological replicates of 10,000 cells per timepoint. *p < 0.05 by two-sided F-test for linear regression versus control during the first 8 minutes of ATP decline.

(c-d) Cells were incubated for 2 hr with both respiratory and glycolytic substrates (10 mM [U-^{13}C]glucose, 5 mM pyruvate). (c) *HK2* knockdown increased the size of serine and glycine metabolite pools. (d) There was only minimal incorporation of glucose carbons into serine in the control group, and this was further decreased by *HK2* knockdown. n = 3 replicates of 500k cells, **p < 0.01, ***p < 0.001 versus non-targeting control, two-way ANOVA with Sidak's multiple comparisons test.

(e) Cells were incubated for 2hr with respiratory substrates (10mM [U-^{13}C]pyruvate) with no 2DG present. There was considerable incorporation of pyruvate carbons into PEP, 3PG and serine in controls, and *HK2* knockdown increased this. n = 3 biological replicates of 500k cells, n = 2 biological replicates of *HK2* PEP, **p < 0.01, ***p < 0.001 versus non-targeting control, two-way ANOVA with Sidak's multiple comparisons test. Data are presented as mean values ±

SEM. Source data, exact p-values, slopes, and 95% confidence intervals are provided as a Source Data file.

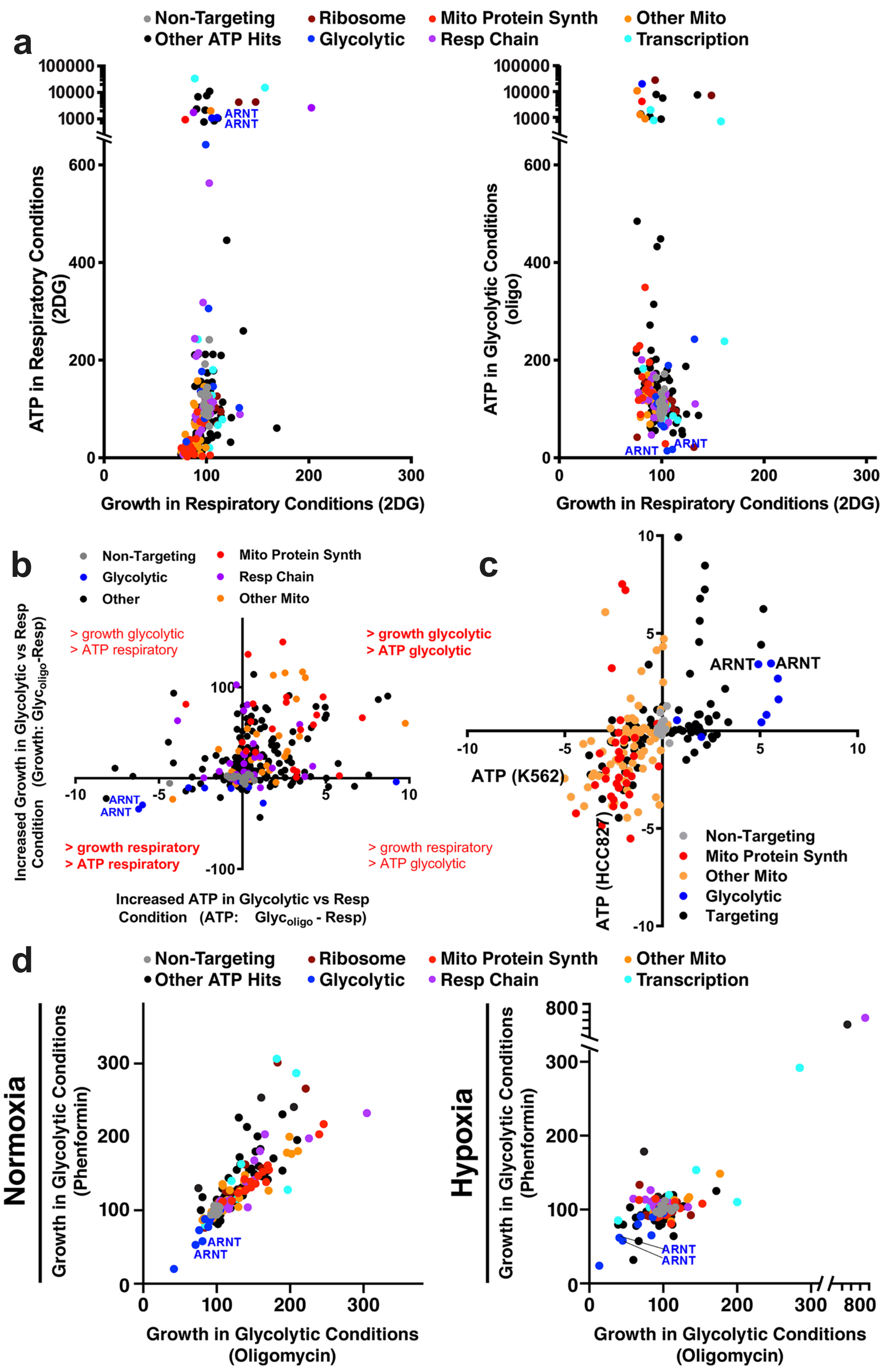

a
Non-Targeting
Other ATP Hits
Ribosome
Glycolytic
Mito Protein Synth
Resp Chain
Other Mito
Transcription
ATP in Respiratory Conditions (2DG)
Growth in Respiratory Conditions (2DG)
ATP in Glycolytic Conditions (oligo)
ARNT
b
Non-Targeting
Glycolytic
Other
Mito Protein Synth
Resp Chain
Other Mito
> growth glycolytic
> ATP respiratory
> ATP glycolytic
> growth respiratory
Increased Growth in Glycolytic vs Resp Condition (Growth: Glyc$_{oligo}$-Resp)
Increased ATP in Glycolytic vs Resp Condition (ATP: Glyc$_{oligo}$ - Resp)
c
ATP (K562)
ATP (HCC827)
Targeting
d
Normoxia
Hypoxia
Growth in Glycolytic Conditions (Phenformin)
Growth in Glycolytic Conditions (Oligomycin)

Figure S6. Impact of high ATP hits on growth and ATP as a function of substrate, Related to Figure 6.

(a) HCC827 cells expressing the CRISPRi mini-library were grown for 3 days in respiratory conditions (10 mM 2DG), and the fold-impact of each sgRNA on growth (mean read count, normalized to nontargeting controls) plotted versus ATP phenotype measured in parallel experiments. Data is from 4 replicates for ATP, and 6 6 for growth. For growth and ATP in respiratory conditions, Non-targeting Pearson r = -0.317, p = 0.200, n = 18; mitochondrial ribosome r = 0.790, p < 0.01, n = 14, p-values for non-zero slope or null hypothesis of no correlation by Pearson's correlation test after removing outliers with ROUT method.

(b) Impact of ATP hit genes on relative growth and ATP in glycolytic versus respiratory conditions. For both respiratory and glycolytic genes, increased growth was associated with increased ATP in that same substrate (13/20 mitochondria-related guides had both increased glycolytic versus respiratory growth and ATP, while 6/20 affected neither ATP nor growth; both ARNT guides had decreased glycolytic versus respiratory growth and ATP). n = 4 replicates of 1M cells for ATP and6 for growth.

(c) ATP phenotypes for HCC827 cells expressing the CRISPRi mini-library (Y-axis) correlated strongly with the corresponding levels in K562 cells expressing the same sgRNA from the full screen (X-axis), both grown in respiratory conditions (Pearson r = 0.510, p < 0.001, n = 202, for targeting guides, r = 0.040, p = 0.883, n = 16, for non-targeting guides, p-values for non-zero slope or null hypothesis of no correlation by Pearson's correlation test). Data from 2 replicates of 1M (mini-library) and 6M (full screen) cells per condition.

(d) HCC827 cells expressing the CRISPRi mini-library were grown in either glycolytic (5 µM oligo) or in an alternate glycolytic substrate (0.5mM phenformin), both in normoxia (left) and hypoxia (1% O_2, right), and the impact of metabolic substrate on growth compared. n = 3 and n = 6 replicates of 1M cells per grou. Source data, exact p-values and 95% confidence intervals are provided as a Source Data file.
