# Supplementary material for: Defining the ATPome reveals cross-optimization of metabolic pathways
Source: Nat Commun. 2020 Aug 28;11:4319. doi: 10.1038/s41467-020-18084-6 (PMC7455733; doi:10.1038/s41467-020-18084-6)
Supplement: Supplementary file 7 — Reporting Summary [file 41467_2020_18084_MOESM7_ESM.pdf]

nature research

Corresponding author(s): Ken Nakamura

Last updated by author(s): Jul 15, 2020

Reporting Summary

Nature Research wishes to improve the reproducibility of the work that we publish. This form provides structure for consistency and transparency in reporting. For further information on Nature Research policies, see our Editorial Policies and the Editorial Policy Checklist.

Statistics

For all statistical analyses, confirm that the following items are present in the figure legend, table legend, main text, or Methods section.

n/a	Confirmed	
☐	☒	The exact sample size (*n*) for each experimental group/condition, given as a discrete number and unit of measurement
☐	☒	A statement on whether measurements were taken from distinct samples or whether the same sample was measured repeatedly
☐	☒	The statistical test(s) used AND whether they are one- or two-sided<br>*Only common tests should be described solely by name; describe more complex techniques in the Methods section.*
☒	☐	A description of all covariates tested
☐	☒	A description of any assumptions or corrections, such as tests of normality and adjustment for multiple comparisons
☐	☒	A full description of the statistical parameters including central tendency (e.g. means) or other basic estimates (e.g. regression coefficient) AND variation (e.g. standard deviation) or associated estimates of uncertainty (e.g. confidence intervals)
☐	☒	For null hypothesis testing, the test statistic (e.g. *F*, *t*, *r*) with confidence intervals, effect sizes, degrees of freedom and *P* value noted<br>*Give P values as exact values whenever suitable.*
☒	☐	For Bayesian analysis, information on the choice of priors and Markov chain Monte Carlo settings
☒	☐	For hierarchical and complex designs, identification of the appropriate level for tests and full reporting of outcomes
☐	☒	Estimates of effect sizes (e.g. Cohen's *d*, Pearson's *r*), indicating how they were calculated

Our web collection on statistics for biologists contains articles on many of the points above.

Software and code

Policy information about availability of computer code

Data collection	Flow cytometry data was collected by BD FacsDiva software (v.8.0). Agilent Seahorse XF96 Analyzer software (v.2.4) was used for oxygen consumption and extracellular acidification rate data collection.
Data analysis	Pre-ranked Gene Set Enrichment Analysis (v.4.0.1) was used to determine enriched pathways and ontology terms among high and low-ATP genes. GraphPad Prism (v.8.0.1) was used for statistical data analysis. FlowJo (v.10.3) was used to analyze flow cytometry data. Agilent Wave software (v.2.4.0.60) was used for Seahorse data analysis. Software for analyzing raw data from deep sequencing is available at https://github.com/mhorlbeck/ScreenProcessing. LC-MS metabolomics data analysis was performed using TraceFinder (v.4.1) in-house R scripts used by the UCLA Metabolomics Center. Western blot images were processed with ImageStudio Lite (v.5.2).

For manuscripts utilizing custom algorithms or software that are central to the research but not yet described in published literature, software must be made available to editors and reviewers. We strongly encourage code deposition in a community repository (e.g. GitHub). See the Nature Research guidelines for submitting code & software for further information.

Data

Policy information about availability of data

All manuscripts must include a data availability statement. This statement should provide the following information, where applicable:

- Accession codes, unique identifiers, or web links for publicly available datasets
- A list of figures that have associated raw data
- A description of any restrictions on data availability

Source data are provided with this paper. All relevant data are available from the authors, and source data are provided as a “Source Data file [https://doi.org/10.7272/Q67S7KZD]”.Source Data file (DOI 10.7272/Q67S7KZD). Software for processing and analysis of sequencing reads from the pooled library was the

same as that used by Gilbert et al., Cell 2014. For the ATP FRET-based screen, phenotypes (average of strongest sgRNAs) for all genes screened in all conditions are provided in Supplementary Data 1, 3, and 4Table S1. Sequences for all sgRNAs were published by Gilbert et al., Cell 2014.

Field-specific reporting

Please select the one below that is the best fit for your research. If you are not sure, read the appropriate sections before making your selection.

☒ Life sciences ☐ Behavioural & social sciences ☐ Ecological, evolutionary & environmental sciences

For a reference copy of the document with all sections, see nature.com/documents/nr-reporting-summary-flat.pdf

Life sciences study design

All studies must disclose on these points even when the disclosure is negative.

Sample size	We predetermined that we would perform at least two replicates of the whole genome and mini-library CRISPRi/a screens, due to design of other published similar whole-genome CRISPR screens (Gilbert et al., Cell 2014), and due to practical limitations. For each replicate, the total cell number collected is calculated based to ensure representation of all CRISPRi/a sgRNA within a given genome-scale or mini-library. For experiments that involved comparison of means between multiple cell lines, sample sizes were chosen based on estimated effect sizes between conditions.
Data exclusions	We preestablished exclusion criteria for CRISPRi/CRISPRa guides if deep sequencing counts fell below a set threshold. Individual CRISPRi/a cell lines that failed to show targeted knockdown upon creation were excluded from validation studies.
Replication	All findings were replicated successfully. All findings were independently replicated at least twice.
Randomization	Allocation of distinct batches of cell subcultures to different metabolic substrate conditions was performed at random.
Blinding	Deep sequencing of CRISPRi/a guides and metabolomics experiments were blinded, as the data was collected by separate lab facilities with coded sample names. For all other experiments, investigators were blinded to group allocation during data collection.

Reporting for specific materials, systems and methods

We require information from authors about some types of materials, experimental systems and methods used in many studies. Here, indicate whether each material, system or method listed is relevant to your study. If you are not sure if a list item applies to your research, read the appropriate section before selecting a response.

Materials & experimental systems

n/a	Involved in the study	
☐	☒	Antibodies
☐	☒	Eukaryotic cell lines
☒	☐	Palaeontology and archaeology
☒	☐	Animals and other organisms
☒	☐	Human research participants
☒	☐	Clinical data
☒	☐	Dual use research of concern

Methods

n/a	Involved in the study	
☒	☐	ChIP-seq
☐	☒	Flow cytometry
☒	☐	MRI-based neuroimaging

Antibodies

Antibodies used	Primary antibodies used recognized human HK1 antibody (ab150423, 1:2500, abcam) and beta-actin antibody (MAB1501R, 1:5000, Millipore Sigma), followed by IRDye 800CW Goat anti-Mouse IgG and IRDye 680RD Goat anti-Rabbit IgG secondary antibodies (Li-cor Biosciences, , 926-32210 and 926-68071 respectively, 1:10000).
Validation	All antibodies were validated for Western blot on manufacturer's websites. HK1 was validated by the manufacturer on human HK1 knockout HEK293T cells, and the beta-actin antibody was validated by the manufacturer on muscle homogenates. We separately validated the HK1 antibody using lysates from human neurons, which had higher expression of HK1 than the K562 cells. Similar to the manufacturer's knockout validation, we used the HK1 antibody to assess knockdown of HK1 in human K562 cells.

Eukaryotic cell lines

Policy information about cell lines

Cell line source(s)	K562 cells were supplied by Jonathan Weissman's lab, and originally supplied by ATCC, catalog CCL-243. HCC827 cells were supplied by ATCC, catalog CCL-2868.

Authentication	None of the cell lines were authenticated beyond authentication methods performed by cell line sources.
Mycoplasma contamination	Cell lines were certified negative for mycoplasma contamination from original sources. Primary patient derived fibroblasts used in our experiments were tested and found to be negative for mycoplasma.
Commonly misidentified lines (See ICLAC register)	No commonly misidentified cell lines were used in these experiments.

Flow Cytometry

Plots

Confirm that:

☒ The axis labels state the marker and fluorochrome used (e.g. CD4-FITC).

☒ The axis scales are clearly visible. Include numbers along axes only for bottom left plot of group (a 'group' is an analysis of identical markers).

☒ All plots are contour plots with outliers or pseudocolor plots.

☒ A numerical value for number of cells or percentage (with statistics) is provided.

Methodology

Sample preparation	The cells used in flow cytometry experiments were K562 cells supplied by the lab of Jonathan Weissman.
Instrument	The instrument used was a BD FacsAria II.
Software	Flow cytometry data was collected by BD FacsDiva software. FlowJo v10 was used to analyze flow cytometry data.
Cell population abundance	Cell populations analyzed were generally >90% positive for BFP if transfected with CRISPRi/a sgRNA and drug-selected, >90% positive for ATP sensor expression due to multiple rounds of selection by flow cytometry, or >90% positive for MitoTracker Green staining.
Gating strategy	Gating strategies are detailed in Figure S1 of our manuscript. First, cells were gated by forward (FSC-A) and side scatter (SSC-A), and then for single cells using FSC-A/FSC-W and SSC-A/SSC-W. Next, cells were gated based on positive BFP fluorescence (compared to non-transfected cells), indicating the presence of a CRISPRi or CRISPRa sgRNA, followed by gating out of cells with donor or FRET signal from the ATP sensor that overlapped with background (too dim) or exceeded the range of the sorter (too bright). The ratio of FRET/Donor was displayed as a histogram, and the top and bottom 25% of cells on this histogram were separated by FACS and collected for sequencing, as described in Mendelsohn et al 2018.<br><br>In order to quantify mitochondrial mass in individual CRISPRi-knockdown and MitoTracker Green-stained lines, FITC/FSC-A was measured after basal condition pre-treatment, with single cell- and positive BFP fluorescence gating performed as in the primary screen.

☒ Tick this box to confirm that a figure exemplifying the gating strategy is provided in the Supplementary Information.
