# Supplementary material for: Defining the ATPome reveals cross-optimization of metabolic pathways
Source: Nat Commun. 2020 Aug 28;11:4319. doi: 10.1038/s41467-020-18084-6 (PMC7455733; doi:10.1038/s41467-020-18084-6)
Supplement: Supplementary file 8 — Description of Additional Supplementary Files [file 41467_2020_18084_MOESM8_ESM.docx]

**Supplementary Data 1. ATP screen data, Related to Figures 1-3.**

Whole genome CRISPRi and CRISPRa screen results, categorized by metabolic substrate (respiratory, glycolytic and basal). ATP FRET phenotypes, z-scores, and p-values are shown for both the ATP FRET sensor and the dead FRET sensor. Genes categorized as robust hits by FRET phenotype (based on a cutoff of 3 standard deviations from the mean ATP phenotype of quasi-genes generated from randomly sampled non-targeting guides) are highlighted yellow, and those with Mann-Whitney two-sided p-values below 0.05 are bolded.

**Supplementary Data 2. Pathway and ontology term enrichment, Related to Figure 2.**

Selected enriched pathways and ontology terms for ATP screen with CRISPRi and CRISPRa perturbation, categorized by metabolic substrate. Enrichment statistics were all calculated by gene set enrichment analysis.

**Supplementary Data 3. Mini-library screen data, Related to Figure 4**

Mini-library enriched with respiratory ATP hit genes screened for impact on mitochondrial content and ATP consumption, and categorized by gene function. Column D represents the mitochondrial content phenotype, which is the log2 fold change in MitoTracker staining normalized to cell size in basal conditions. For ATP consumption, columns E-G represent the ATP depletion phenotype, which is the log2 fold change in ATP phenotype of cells incubated in respiratory, glycolytic, or basal conditions before versus after ATP production was blocked. Column I is the specific sgRNA identifier.

**Supplementary Data 4. ATP and growth phenotypes of cells expressing CRISPRi mini-library, related to Figures 6, S6.**

Table shows the impact of the ATP respiratory hit-enriched CRISRPi mini-library on ATP levels in respiratory and glycolytic conditions, as well as on growth in respiratory (2DG) and glycolytic (oligo and phenformin) substrates. Cell growth is shown in both normoxia and hypoxia, as well as the impact of hypoxia on growth in basal conditions.
