# Supplementary material for: Defining the ATPome reveals cross-optimization of metabolic pathways
Source: Nat Commun. 2020 Aug 28;11:4319. doi: 10.1038/s41467-020-18084-6 (PMC7455733; doi:10.1038/s41467-020-18084-6)
Supplement: Supplementary file 2 — Peer Review File [file 41467_2020_18084_MOESM2_ESM.docx]

Peer Review Information

**Journal:** Nature Communications

**Manuscript title:** Defining the ATPome Reveals Cross-Optimization of Metabolic Pathways

**Corresponding author name(s):** Dr Ken Nakamura

**Editorial notes:**

**Transferred manuscripts**

This manuscript has been previously reviewed at another journal that is not operating a transparent peer review scheme. This document only contains reviewer comments and rebuttal letters for versions considered at *Nature Communications*.

**Reviewer comments & decisions:**

**Reviewer comments, first version:**

Reviewer #1 (Remarks to the Author):

The authors previously developed a high throughout screen integrating the CRISPRi/CRISPRa screen and an ATP biosensor to identify novel ATP-regulatory proteins. In the current study, the authors employed this system for a whole genome screen for genetic regulators of ATP under three metabolic conditions: respiration, glycolysis and basal conditions. The investigations on which genes are predominant ATP drivers/consumer under respiration or glycolysis condition are very interesting. In addition, the fact that the authors incorporated both loss-of (CRISPRi) and gain-of (CRISPRa) function approaches into their screening allows the cross verification of the screening results, increasing the credibility of the ATP-regulatory hits.

However, there are serious concerns about the experimental designs of the metabolic conditions. The authors created a respiration condition by the addition of 2DG for blocking glycolysis and supplied the cells with pyruvate as a source for respiration. Aside from glucose and pyruvate, it has been well-established that glutamine is a critical carbon source to support the biosynthesis, energy production and growth of cells. However the level of glutamine is not taken into account when establishing the respiration condition. For the glycolysis condition, beside oligomycin (in inhibitor of mitochondrial respiration), the author mentioned the use of low dose (3mM) of 2DG to mimic the glycolysis condition (Line 109). However, 2DG at 3mM has readily lowered the ATP level in cells, most likely due to glycolysis inhibition. Thus, the authors likely ended up analyzing the conditions with inhibited respiration and defective glycolysis. Overall, the metabolic conditions that the authors used for the screening could merely an artificial condition, which do not reflect physiological or pathological situations. The significance of this study is thus greatly diminished. Aside from the above, there are several issues as noted below:

Specific comments
1. The authors primarily used K562, a leukemia cells, to screen for the ATP regulators. However, the authors switched to HCC827 to study the impact of the identified ATP regulators on cell growth. HCC827 is a lung cancer cell line which has very different biology and genetics from the K562 leukemia cells. How could the authors reconcile the observations from K562 and HCC827 cells? The author should provide experimental evidence showing the generality of the identified ATP hits in different cell lines to substantiate their notions.

2. Besides glycolysis inhibition, 2DG has been shown to activate multiple prosurvival pathways such as PI3K/AKT (Zhong et al JBC 2009.p23225). In view of the important function of Akt in promoting glucose uptake and HK2 activation, it is hard to evaluate whether authors’ observation on HK2 as an ATP consumer is a direct consequence of glycolysis inhibition or due to the non-specific effects.

3. HK2 is known to phosphorylate glucose and 2DG but not metabolize 2DG. The statement in line 279-280 should be corrected.

4. To verify if HK2-mediated phosphorylation of glucose/2DG accounts for ATP consumption, the author should directly measure the ATP level in cells before and after the reaction under the three metabolic conditions.

5. The authors stated that inhibiting the transcriptions factors that promote glycolysis including HIF1 and Myc, boosted up mitochondrial-deprived ATP, and this effect was phenocopied by inhibiting of glycolytic enzymes. While Myc has been shown to regulate glycolysis, its role and regulation in OXPHOS is equally if not more profound. The author should amend the statements.


Reviewer #2 (Remarks to the Author):

In this study, the authors performed a genome-wide screen to identify genes responsible for elevating or repressing cellular ATP levels as read out using a FRET-based sensor. This study builds on past work from this group who performed a similar analysis focused only on mitochondrial genes, and reflects a clever utilization of an elegant system to ask a more general question. In general, it is an interesting data set and should be of broad interest. Nevertheless, this address a very complex question and assessment of ATP production and consumption is challenging using ATP level as a primary read out. While they do discuss some controls for other ways a hit might change ATP levels, some conclusions may be overreaching and the authors might consider several points when interpreting their data.

1. The conclusions they draw are broad (perhaps too broad), and the authors spend a lot of time discussing the specific genes and their corresponding pathways that modulate their FRET signal in “basal”, “respiration”, and “glycolytic” states as defined by treatment with oligomycin or 2DG+pyruvate. In the context of how these findings are presented, the most compelling evidence is the definition of the cellular “ATPome” in terms of identifying genes that may directly or indirectly contribute to ATP levels, although not all will reflect direct changes in consumption or production. First, it is hard with a genetic perturbation to determine what is a direct effect, and what represents cellular adaptation. The experiment to track acute changes in ATP with perturbation is a nice way to infer consumption, but many things may be changing to adapt to the perturbation, it is hard to make quantitative statements about what accounts for the most ATP production or consumption.

2. Some of the claims in this paper should rewritten as to not overstate their novelty. There exists an extensive biochemical literature on what constitutes cellular ATP production and consumption. While the authors take an exhaustive genetic approach to answering the question, many of their findings actually validate known observations from previous studies. For example, Buttgereit and Brand accounted for the major consumers of ATP in the cell in 1995 using biochemical methods (PMC1136240). In their study, Buttgeriet and Brand identified RNA and protein synthesis as major consumers of ATP. One could interpret the fact that high ATP CRISPRi hits included factors that regulate transcription, translation, and protein degradation as a genetic validation of what is previously biochemically defined. Accordingly, the authors even use cycloheximide as a positive control to elevate ATP levels in the cells. At the very least, the authors should cite this work. Additionally, the findings that inhibiting HK2 elevates cellular ATP through inhibiting the “investment phase” of glycolysis has also been previously identified (PMC6062487). Nonetheless, the fact that the authors were able to identify these pathways in their screen speaks to the quality of the study and the overall credibility of the results of their screen.

3. Cell size is included as a control in Figure 4, but otherwise is not considered as another variable that would affect ATP concentration (and therefore FRET signal). Also, as they point out energy reflected in the ratio of ATP to ADP and AMP, and just because Adenylate kinase did not score in their screen does not mean all hits that affect ATP levels affect energy charge in the same way. At a minimum this limitation of the analysis should be considered and discussed.

4. The authors should clarify what they measuring in terms of respiration in different experiments. Maximal respiration (uncoupled respiration) is stated in some places, and simply respiration in others. Baseline respiration should be reported in all cases, and when appropriate maximal respiration can also be reported. However, it is not clear what a change in maximal respiration means with their hits, as uncoupled respiration is by definition uncoupled from ATP production and if hits change ATP without affecting basal respiration something more surprising is going on.

5. The paper is presented in a way that may be inaccessible to a general audience. More explanation is needed for the experimental conditions and the rationale behind such decisions behind the experimental design should also be clarified. Rewriting the main text in a way to become more accessible and less overstated would greatly increase the impact of the work.


Reviewer #3 (Remarks to the Author):

Summary: Bennet et al. perform a genome wide screen using both CRISPRi and CRISPRa to comprehensively characterize genes that regulated cellular ATP. By culturing cells in conditions to promote energy production exclusively by glycolysis or oxidative respiration as well as in basal conditions, the authors are able identify targets which regulate ATP levels when cells are preferentially using particular substrates in addition to those that promote supraphysiological levels of ATP. By taking a genome wide approach as well as using strategies to either reduce (CRISPRi) or promote (CRISPRa) target gene expression, the authors generate a comprehensive overview of the metabolic regulation of ATP levels. Additionally, key findings are confirmed in other cell lines suggesting that results from this screen may be broadly applicable to diverse cell types and across tissue of origin.

Overall, the manuscript in its current form presents a well thought out and carefully designed study. Results from this study confirm many ideas that have not been formally proven about energy metabolism and regulation while providing new insight into ATP regulation. The study could be further strengthened by focusing on a single hit or prominent pathway and providing greater mechanistic insight into how modulation of these genes precisely regulate ATP availability and in which biological contexts this is most relevant (i.e. disease state, developmental program etc.).

1. While the current study is interesting and the data is compelling, the manuscript largely reads as just an overview of the results of the screen with commentary from the literature about known gene function and relevance. In a few areas the authors go a bit further to test a handful of hits in a limited capacity:
a. Requirement of an intact respiratory chain for some high ATP hits to boost respiration (Figure 4)
b. H2K as a major ATP consumer and overall regulator of ATP pool (Figure 5)
c. High ATP hits promote tumor cell growth (Figure 6)
More mechanistic insight on any one of these would greatly enhance the study.
1. Does H2K channel more glucose through the PPP over glycolysis versus other isoforms(C13 Tracing, and metabolic flux analysis)? – related to figure 5
2. Are the growth promoting high ATP hits functionally relevant to tumor growth in vivo? What do clinical outcomes look for patients that have high/low expression of genes related to ATP high vs low state? Do hits from the screen faithfully predict the effects on tumor growth for use of available metabolic inhibitors? Does successful implementation require combination therapies to force cellular reliance on particular substrates (glycolysis vs respiration)? – related to Figure 6

Minor:
1. The addition of a few schematics providing an over view of how particular pathways related to ATP and energy regulation would be helpful and improve readability – HK2, HIF pathway and related genes etc.

**Author rebuttal, first version:**

Reviewer #1 (Remarks to the Author):

The authors previously developed a high throughout screen integrating the CRISPRi/CRISPRa screen and an ATP biosensor to identify novel ATP-regulatory proteins. In the current study, the authors employed this system for a whole genome screen for genetic regulators of ATP under three metabolic conditions: respiration, glycolysis and basal conditions. The investigations on which genes are predominant ATP drivers/consumer under respiration or glycolysis condition are very interesting. In addition, the fact that the authors incorporated both loss-of (CRISPRi) and gain-of (CRISPRa) function approaches into their screening allows the cross verification of the screening results, increasing the credibility of the ATP-regulatory hits.

However, there are serious concerns about the experimental designs of the metabolic conditions. The authors created a respiration condition by the addition of 2DG for blocking glycolysis and supplied the cells with pyruvate as a source for respiration. Aside from glucose and pyruvate, it has been well-established that glutamine is a critical carbon source to support the biosynthesis, energy production and growth of cells. However the level of glutamine is not taken into account when establishing the respiration condition.

We thank the reviewer for the feedback on this important point. We designed our screen to force cells to depend entirely on either respiration (respiratory conditions) or glycolysis (glycolytic conditions) for ATP, or to let cells use either. As such, the respiratory and glycolytic conditions intentionally create metabolic extremes, enabling us to identify the widest possible range of ATP regulating genes and pathways, and to provide the most robust possible data. Moreover, pyruvate is an appropriate respiratory substrate for central focus in this manuscript, given that it's a key energy metabolite linking glycolysis to respiration. As such, although cells were grown in media containing glutamine, we did not include glutamine in the acute metabolic treatments used in our whole genome screens. Importantly, using pyruvate as substrate we identified numerous components of the electron transport chain and most mitochondrial ribosomal proteins that are essential for mitochondrial-derived ATP production, proving the sensitivity of our approach to identify common components of the respiratory chain that are critical independent of the respiratory substrate. In addition, we also identified enzymes specifically responsible for pyruvate metabolism (e.g. both PDHA1 and PDHB had very strong low ATP phenotypes in respiratory conditions).

However, we fully agree with the reviewer that additional energy substrate-specific ATP regulating genes would be identified using different energy substrates, and elucidating these will be of great interest, as we now state in the manuscript. Glutamine is indeed of great interest, and we have now performed additional experiments examining the impact of glutamine rather than pyruvate as the respiratory substrate on ATP levels in a mitochondrial-gene enriched CRISPRi mini-library (Mendelsohn et al., *PLoS Biol*, PMID 30148842) and observed high similarity with our prior findings where pyruvate was substrate (new Figure S1b).

Ultimately, it will also be important to investigate different “physiologic” and “pathologic” substrate conditions to determine which of the ATP regulating hits are most critical specifically in each setting. However, this composition is rarely understood, and also differs between tissue types and in pathologic conditions.

For the glycolysis condition, beside oligomycin (in inhibitor of mitochondrial respiration), the author mentioned the use of low dose (3mM) of 2DG to mimic the glycolysis condition (Line 109). However, 2DG at 3mM has readily lowered the ATP level in cells, most likely due to glycolysis inhibition. Thus, the authors likely ended up analyzing the conditions with inhibited respiration and defective glycolysis. Overall, the metabolic conditions that the authors used
for the screening could merely an artificial condition, which do not reflect physiological or pathological situations. The significance of this study is thus greatly diminished.

The reviewer raises an important point. One of our central observations is that it is much easier to restore ATP levels back to baseline rather than to raise them to supraphysiologic levels. Therefore, in order to identify the broadest spectrum of ATP-regulating genes and pathways, it was critical that we include conditions in which the cells must raise their ATP levels back towards baseline using either respiration or glycolysis. However, because many tumor cell lines rely heavily on glycolysis, fully inhibiting respiration alone did not substantially decrease ATP in the cells over the time course studied. However, with respiration fully blocked, addition of low dose 2DG caused a partial decrease in ATP, enabling us to robustly detect genes that either prevented the ATP decrease (high ATP hits) or dropped ATP to a greater extent (low ATP hits). Importantly, we showed in our prior manuscript that ATP production under this condition (oligomycin and low dose 2DG) is still derived entirely from glycolysis (Mendelsohn et al., *PLoS Biol*, PMID 30148842), and we have now clarified this in the text. In the present manuscript, untargeted metabolomics demonstrate that glycolytic metabolites are detectable when 2DG is included in these conditions (Figure 5), and there is no significant difference in labeled glucose conversion into lactate (Figure A).

**Figure A. Glycolysis increases in response to respiratory inhibition (oligomycin) despite the presence of low dose 2DG.**

**Left:** K562 cells were treated for 2 hours with glycolysis-only substrates 1) 2mM [U-^13^C]glucose (black), 2) 2mM [U-^13^C]glucose, 5 μM oligomycin and a low dosage of 3 mM 2DG (blue), or 3) 2mM [U-^13^C]glucose and 5 μM oligomycin (red). Inhibiting respiration with oligomycin increased the size of glycolytic metabolite pools, even in the presence of low dose 2DG.

**Right:** Oligomycin used in the ATP screen glycolytic conditions also increases glucose-derived carbon incorporated into lactate. The low dose of 2DG does not significantly impact this incorporation. Data are means ± SEM, n = 3 samples, 500k cells/sample, **p < 0.01, ***p < 0.001 versus non-targeting control, two-way ANOVA with Sidak’s multiple comparisons test.

Importantly, the hits identified in these substrate conditions also confirm the validity of our paradigm (e.g. mitochondrial respiratory genes were high ATP hits in glycolytic conditions, but low ATP hits in respiratory conditions [Mendelsohn et al., and Figure 2 in the current manuscript]). Moreover, we also assessed the entire genome in a third substrate “basal” condition, in which both glycolysis and respiration were enabled in the absence of any 2DG. Indeed**, the full scope of substrates and gene manipulations (CRISPRi and CRISPRa) is a major strength of our work**, defining the ATPome in an extremely comprehensive manner. As described in the response to the preceding question, our goal was not to precisely mimic physiologic or pathologic substrates, which have generally not been defined, as this will be an important objective of future studies. But our comprehensive analysis has both great biologic significance for our understanding of metabolic control of energy levels, and we believe will also be of great importance in understanding the role of energy failure in disease, and how it may be targeted therapeutically. Indeed, **we emphasize that our analysis has already identified numerous genes believed to produce disease through effects on energy failure across a range of metabolically vulnerable cell and tissue types.**

Aside from the above, there are several issues as noted below:

1. The authors primarily used K562, a leukemia cells, to screen for the ATP regulators. However, the authors switched to HCC827 to study the impact of the identified ATP regulators on cell growth. HCC827 is a lung cancer cell line which has very different biology and genetics from the K562 leukemia cells.

How could the authors reconcile the observations from K562 and HCC827 cells? The author should provide experimental evidence showing the generality of the identified ATP hits in different cell lines to substantiate their notions.

We agree with the reviewer that experimental evidence showing the generality of identified ATP hits in different cell lines is important to support the roles of these genes in regulating metabolism. In Figures 4 and S2, we demonstrate that several of our high-ATP hits with knockdown in K562 leukemia cells produce similar ATP phenotypes in fibroblast lines, and that knockdown of these hits increased oxygen consumption rate. In the HCC827 cells, we screened a mini-library enriched with gene knockdowns with both high- and low-ATP phenotypes. Moreover, we did indeed measure robust concordance between ATP phenotypes in K562 and HCC827 cells following gene knockdown, included as new panel Figure S5c. These findings support our general observation and hypothesis that most cells have similar bioenergetic functions when forced to rely on the same substrate, although identifying cell type-specific regulators is an important area for ongoing investigation.

2. Besides glycolysis inhibition, 2DG has been shown to activate multiple prosurvival pathways such as PI3K/AKT (Zhong et al JBC 2009.p23225). In view of the important function of Akt in promoting glucose uptake and HK2 activation, it is hard to evaluate whether authors’ observation on HK2 as an ATP consumer is a direct consequence of glycolysis inhibition or due to the non-specific effects.

We thank the reviewer for drawing attention to the Zhong et al. manuscript, and agree that it is important to consider possible non-specific effects of metabolic inhibitors. However, these effects are very unlikely to explain our findings with HK2 as a major regulator of ATP consumption, as we observed that HK2 knockdown slows ATP consumption in conditions where 2DG was present only during ~15 minutes of ATP depletion (in basal conditions in Figure 5, now Figure 4d and S4a). In contrast, Zhong et al. examined the impact of 2DG treatment over 4 hours, except in the context of ERK phosphorylation (their Figure 3), where they saw only a very small increase in phosphorylation over 15 minutes. Moreover, we also show that HK2 knockdown decreases ATP consumption in basal conditions where glucose is present, but 2DG is absent (in Figure 5, now Figure S4b, basal conditions).

Notably, we agree that the interaction between Akt and HK2 could indeed contribute to the HK2-mediated impact on ATP levels. Akt phosphorylates HK2 and facilitates its interaction with mitochondria in the absence of 2DG (Roberts, Miyamoto, *Cell Death Diff* 2015, PMID 25578149). John et al. (John et al., *PLOS One* 2011, PMID 21408025) also showed that HK2 dissociates from the mitochondria after 20 minutes of glucose removal, and this is blocked by overexpression of constitutively activated Akt. Therefore, the effect of Akt on HK2 could contribute to the ATP phenotype of HK2 knockdown in our respiration-only condition, although the impact on ATP would likely be a specific consequence of glycolysis inhibition by glucose deprivation, rather than a non-specific effect.

3. HK2 is known to phosphorylate glucose and 2DG but not metabolize 2DG. The statement in line 279-280 should be corrected.

Thank you for pointing out this error, which we have corrected.

4. To verify if HK2-mediated phosphorylation of glucose/2DG accounts for ATP consumption, the author should directly measure the ATP level in cells before and after the reaction under the three metabolic conditions.

We agree that it is important to establish if HK2-mediated phosphorylation is a major contributor to ATP consumption. We believe what the reviewer is proposing is exactly what we presented in Figure 5 (now Figure 4d and S4a,b), where we compared the impact of knocking down HK2 (versus other key genes and control) on ATP consumption rates in K562 cells, under the three metabolic conditions used in our screen, as well as in the presence of an alternative glycolytic inhibitor. We have also added new data to the manuscript demonstrating that HK2 knockdown decreases the size of PPP metabolite pools and decreases glucose-derived carbon incorporation into PPP metabolite pools (Figure 5), to a greater extent than HK1 knockdown. As such, our data suggest that HK2 is a major regulator of ATP consumption in the presence of glucose, both because of its direct role in phosphorylation, and because of its specific role in diverting glucose away from glycolysis into the (energy consuming) PPP.

5. The authors stated that inhibiting the transcriptions factors that promote glycolysis including HIF1 and Myc, boosted up mitochondrial-deprived ATP, and this effect was phenocopied by inhibiting of glycolytic enzymes. While Myc has been shown to regulate glycolysis, its role and regulation in OXPHOS is equally if not more profound. The author should amend the statements.  

We thank the reviewer for drawing attention to this important aspect of Myc biology. We agree that in addition to its roles in regulating glycolysis, Myc also has important roles in the regulation of OXPHOS and the interplay between these roles remains to be fully elucidated, especially under the metabolic conditions examined in our screen. As such, we have removed Myc from this statement.

Reviewer #2 (Remarks to the Author):

In this study, the authors performed a genome-wide screen to identify genes responsible for elevating or repressing cellular ATP levels as read out using a FRET-based sensor. This study builds on past work from this group who performed a similar analysis focused only on mitochondrial genes, and reflects a clever utilization of an elegant system to ask a more general question. In general, it is an interesting data set and should be of broad interest. Nevertheless, this address a very complex question and assessment of ATP production and consumption is challenging using ATP level as a primary read out. While they do discuss some controls for other ways a hit might change ATP levels, some conclusions may be overreaching and the authors might consider several points when interpreting their data.

1. The conclusions they draw are broad (perhaps too broad), and the authors spend a lot of time discussing the specific genes and their corresponding pathways that modulate their FRET signal in “basal”, “respiration”, and “glycolytic” states as defined by treatment with oligomycin or 2DG+pyruvate. In the context of how these findings are presented, the most compelling evidence is the definition of the cellular “ATPome” in terms of identifying genes that may directly or indirectly contribute to ATP levels, although not all will reflect direct changes in consumption or production. First, it is hard with a genetic perturbation to determine what is a direct effect, and what represents cellular adaptation. The experiment to track acute changes in ATP with perturbation is a nice way to infer consumption, but many things may be changing to adapt to the perturbation, it is hard to make quantitative statements about what accounts for the most ATP production or consumption.

We appreciate the reviewer’s thoughtful perspective and agree it is important to consider which genes have direct effects on metabolism, and which represent cellular adaptation. Indeed, a key feature of our experimental design is that we expose cells to the glycolytic or respiratory conditions just before imaging, so as to enrich for genes that have primary effects on regulating ATP. Most of our hits also disrupt ATP levels in specific substrate conditions, also supporting a primary rather than non-specific effect on ATP. This is further supported by our prior identification of ≈ 85% of mitochondrial ribosomal proteins as low ATP respiratory hits, while none of these were low ATP hits in glycolytic conditions (Mendelsohn et al., *PLoS Biol*, PMID 30148842). Nonetheless, the reviewer is absolutely correct that a subset of our hits likely act through more indirect mechanisms. In particular, some low ATP hits likely decrease ATP through non-specific mechanisms. In contrast, we suspect this is quite rare among high ATP hits, as non-specific toxicity seems less likely to boost ATP levels. Moreover, while we agree that other mechanisms such as cellular adaptation likely underlie some of our hits, we also believe that defining such cellular adaptations is important to understanding how metabolism controls ATP levels, and how energy levels can be targeted therapeutically.

With regard to the role of adaptation in ATP consumption, as the reviewer points out, we’ve tried to minimize this by measuring the rate of ATP consumption soon after inhibiting ATP synthesis, while validating the effects with cycloheximide to block protein synthesis. However, we agree that genes with the greatest impact on ATP consumption likely involve an array of factors. As such, we’ve been careful to qualify our conclusions about HK2 as a major regulator (rather than sole driver) of energy consumption in our paradigm. In addition, we’ve used metabolomics to further delineate how HK2 knockdown influence ATP metabolism. New Figure 5 shows direct effects of HK2 knockdown including decreased size of PPP metabolite pools and decreased incorporation of glucose carbons into PPP metabolites, to a greater extent than HK1 knockdown. The PPP catabolizes glucose and consumes ATP, presumably contributing to the high ATP levels and low ATP consumption in glycolytic and basal conditions with HK2 knockdown. HK2 knockdown also increased serine metabolite pool size and increased incorporation of pyruvate carbons into serine, an energy consuming process that likely contributes to the high ATP consumption we observed in respiratory conditions.

2. Some of the claims in this paper should rewritten as to not overstate their novelty. There exists an extensive biochemical literature on what constitutes cellular ATP production and consumption. While the authors take an exhaustive genetic approach to answering the question, many of their findings actually validate known observations from previous studies. For example, Buttgereit and Brand accounted for the major consumers of ATP in the cell in 1995 using biochemical methods (PMC1136240). In their study, Buttgeriet and Brand identified RNA and protein synthesis as major consumers of ATP. One could interpret the fact that high ATP CRISPRi hits included factors that regulate transcription, translation, and protein degradation as a genetic validation of what is previously biochemically defined. Accordingly, the authors even use cycloheximide as a positive control to elevate ATP levels in the cells. At the very least, the authors should cite this work.

Additionally, the findings that inhibiting HK2 elevates cellular ATP through inhibiting the “investment phase” of glycolysis has also been previously identified (PMC6062487). Nonetheless, the fact that the authors were able to identify these pathways in their screen speaks to the quality of the study and the overall credibility of the results of their screen.

Thank you for pointing out these important articles, which we have now cited in the discussion. We agree that it has been established that RNA and protein synthesis are important consumers of ATP, and that inhibiting HK2 can increase ATP by inhibiting the initial investment phase of glycolysis, and these prior findings help validate our findings. However, we also emphasize that the novelty of our study is both in comparing the ATP consumption of different classes of genes, and in interrogating the relative contributions of different genes within a given class to these processes, including different isoforms of the same gene, or different proteins within the same complex. In particular, we identify HK2 as a major substrate-dependent regulator of ATP consumption in our paradigm. These are areas in which there is very little published information. We have reviewed the manuscript to try to ensure our claims do not overstate their novelty.

3. Cell size is included as a control in Figure 4, but otherwise is not considered as another variable that would affect ATP concentration (and therefore FRET signal).

One advantage of the ATP FRET sensor over luciferase assays for estimating relative ATP is the FRET sensor measurements are theoretically independent of cell size. We now present data from two independent experiments sorting a CRISPRa sublibrary, both of which show no significant correlation between cell size and FRET(new Figure S1a).

Also, as they point out energy reflected in the ratio of ATP to ADP and AMP, and just because Adenylate kinase did not score in their screen does not mean all hits that affect ATP levels affect energy charge in the same way. At a minimum this limitation of the analysis should be considered and discussed.

This is an important point. We believe our finding that key enzymes that regulate the balance between ATP, ADP and AMP were not identified as hits, combined with our identification of known regulators of respiration (e.g. mitochondrial ribosomal proteins) and glycolysis (e.g. ARNT, HIF1a) strongly supports that our ATP hits are truly regulating ATP production and consumption, not simply influencing the interconversion between these metabolites. However, we agree with the reviewer that we do not know the scope of how different hits impact energy charge. This is an important area for investigation, which we now acknowledge and discuss in the manuscript.

4. The authors should clarify what they measuring in terms of respiration in different experiments. Maximal respiration (uncoupled respiration) is stated in some places, and simply respiration in others. Baseline respiration should be reported in all cases, and when appropriate maximal respiration can also be reported. However, it is not clear what a change in maximal respiration means with their hits, as uncoupled respiration is by definition uncoupled from ATP production and if hits change ATP without affecting basal respiration something more surprising is going on.

Thank you for pointing this out. In the initial submission, we reported basal respiration and respiration following oligomycin (i.e. respiration not contributing to ATP production) plotted against extracellular acidification (a surrogate of glycolysis) in Figure S3d. However, the original text did not specify where basal respiration was measured, or discuss the results. We have corrected this, and now highlight basal respiration together with maximal respiration in Figure 4a,b.

Importantly, we believe that the measurements of maximal respiration are directly relevant to interpreting our hits. Maximal respiration provides an estimate of a cell’s maximal capacity to make ATP, with the difference between maximal and basal respiration providing an estimate of the added capacity of cells to make ATP beyond the basal condition (i.e. spare respiratory capacity), and is thus of importance in assessing whether high ATP hits enable cells to increase ATP production. Experimentally, the primary screens were done in K562 cells including with restricted substrate, whereas the Seahorse assessments were performed in fibroblasts which are inherently quiescent with low basal respiration. As such, examining basal and maximal respiration together provides a more comprehensive assessment of a cell’s capacity to produce mitochondrial-derived ATP. However, ultimately, we found that the high ATP hits increased both basal and maximal respiration in control fibroblasts.

5. The paper is presented in a way that may be inaccessible to a general audience. More explanation is needed for the experimental conditions and the rationale behind such decisions behind the experimental design should also be clarified. Rewriting the main text in a way to become more accessible and less overstated would greatly increase the impact of the work.

Thank you for this comment. We have worked with a scientific editor to ensure the main text is accessible, and (as discussed above) to avoid overstating while still communicating the novelty and significance of the findings. As there are significant space constraints that limit many interesting points we’d like to make, we’ve tried to communicate our rationale and findings in the most efficient way possible.

Reviewer #3 (Remarks to the Author):

Summary: Bennet et al. perform a genome wide screen using both CRISPRi and CRISPRa to comprehensively characterize genes that regulated cellular ATP. By culturing cells in conditions to promote energy production exclusively by glycolysis or oxidative respiration as well as in basal conditions, the authors are able identify targets which regulate ATP levels when cells are preferentially using particular substrates in addition to those that promote supraphysiological levels of ATP. By taking a genome wide approach as well as using strategies to either reduce (CRISPRi) or promote (CRISPRa) target gene expression, the authors generate a comprehensive overview of the metabolic regulation of ATP levels. Additionally, key findings are confirmed in other cell lines suggesting that results from this screen may be broadly applicable to diverse cell types and across tissue of origin. 

Overall, the manuscript in its current form presents a well thought out and carefully designed study. Results from this study confirm many ideas that have not been formally proven about energy metabolism and regulation while providing new insight into ATP regulation. The study could be further strengthened by focusing on a single hit or prominent pathway and providing greater mechanistic insight into how modulation of these genes precisely regulate ATP availability and in which biological contexts this is most relevant (i.e. disease state, developmental program etc.). 

1. While the current study is interesting and the data is compelling, the manuscript largely reads as just an overview of the results of the screen with commentary from the literature about known gene function and relevance. In a few areas the authors go a bit further to test a handful of hits in a limited capacity: 
a. Requirement of an intact respiratory chain for some high ATP hits to boost respiration (Figure 4)
b. H2K as a major ATP consumer and overall regulator of ATP pool (Figure 5)
c. High ATP hits promote tumor cell growth (Figure 6)
More mechanistic insight on any one of these would greatly enhance the study.

1. Does H2K channel more glucose through the PPP over glycolysis versus other isoforms(C13 Tracing, and metabolic flux analysis)? – related to figure 5

We thank the reviewer for recognizing the novelty, breadth and experimental rigor of our work. We agree that a critical next step is to investigate individual genes and pathways identified. Our current manuscript begins this process at a systems level, stratifying genes by the mechanisms by which they increase ATP. Specifically, we develop new assays to assess energy consumption, and distinguish it from energy production, and also assay the impact on mitochondrial content, and use fibroblasts from Leigh syndrome patients to show that the capacity of high ATP hits to boost energy production depends on the mechanism by which ATP is compromised. As suggested, we’ve now focused on HK2, which we identified as particularly critical in regulating ATP consumption and boosting ATP levels across substrates.

We now show metabolomic data that contribute to the ATP level and ATP consumption phenotypes seen with HK2 knockdown. Specifically, we show that HK2 knockdown decreased the size of PPP metabolite pools and decreased incorporation of glucose carbons into PPP metabolites, while HK1 knockdown produced less or no effect on several of the same metabolites (Figures 5, S4). These data, along with our observation that PGD knockdown increased ATP in glycolytic conditions and slowed ATP consumption under basal conditions (Figure 3, 4d), identify HK2 regulation of the PPP as a major, systems-wide contributor to ATP metabolism.

Interestingly, in respiratory conditions, we found that HK2 knockdown influenced both energy consuming and producing pathways. Specifically, HK2 knockdown increased the size of serine pools, and increased incorporation of pyruvate carbons into serine. This gluconeogenic process presumably consumes energy, and contributes to increased ATP consumption in respiratory conditions (Figure 4). However, we also observed that HK2 knockdown increased the size of TCA metabolite pools, and increased incorporation of pyruvate carbons into these metabolites. These data, together with our observation that HK2 knockdown increased basal and maximal OCR in healthy and respiration-deficient patient fibroblasts, supports that HK2 knockdown may increase respiration by increasing substrate availability. As such, our data suggest that HK2 influences ATP level by regulating both metabolic substrate availability and the activity of energy-consuming pathways.

2. Are the growth promoting high ATP hits functionally relevant to tumor growth in vivo

This is our hypothesis, and an important question. Although addressing this experimentally is well beyond the scope of this manuscript, our data provide compelling evidence that metabolic substrate including O_2_ level strongly influence tumor growth. We also point out that metabolic substrate may differ greatly between tumor types and between primary tumors and their metastases, therefore defining the range of impacts of changes in gene expression as a function of substrate is critical.

What do clinical outcomes look for patients that have high/low expression of genes related to ATP high vs low state?

This is an important question and we agree with the reviewer that considering clinical outcomes for patients with differences in expression of hit genes might contribute to understanding the functional consequences of high or low ATP. Currently, clinical outcomes such as survival cannot be reliably correlated to expression of genes related to ATP high vs. low state. Our data indicates that gene effects on ATP are highly substrate dependent, and the substrate environment of tumors is not discernible from routine clinical imaging or tests. Tumors can vary in substrate availability and oxygenation even in a given patient, however these details are not measured or reported in standard clinical documentation. In addition, there is great genetic and locational heterogeneity across tumor types and within patients themselves (different tumors in different locations receiving different treatments, in addition to nutritional states of patients). A retrospective analysis involving highly heterogeneous and uncontrolled variables would be highly confounded and likely fail to answer the question.

Do hits from the screen faithfully predict the effects on tumor growth for use of available metabolic inhibitors?

We are very interested in this question, but to our knowledge this has not been tested in a systematic way, either in rodent models or in patients. The available metabolic inhibitors used with greatest clinical experience include 2DG and metformin, neither of which have become standard in cancer regimens. Metformin requires expression of organic cation transporters that are variably expressed in tumor cells, which likely contributed to inconsistent effects and raised questions concerning its broader applicability (Checkley et al., *Cancer Prev Res*, PMID 28154203). Glycolysis inhibition with 2DG has been evaluated as a potential therapy in tumors, for example non-small cell lung cancer (Liu et al., *Lung Cancer*, PMID 30089593). However, 2DG is a limited approach that targets glycolysis only and has not been found to have consistent activity as a single agent, and it remains to be determined how glycolytic inhibition can be integrated into existing lung cancer treatment paradigms. As such, the extent of metabolic inhibition achieved in tumors is unclear, and neither inhibitor has been characterized across tumors with specific metabolic phenotypes. Our data suggest multiple potential strategies, one being that tumors specifically deficient in meeting ATP requirements by oxidative phosphorylation would be sensitized to 2DG. Another implementation strategy is pharmacologically manipulating ATP state in the context of forced reliance on a specific metabolic state to generate *synthetic lethality*. These approaches will be investigated in future studies.

Does successful implementation require combination therapies to force cellular reliance on particular substrates (glycolysis vs respiration)? – related to Figure 6

This is our hypothesis, and we explicitly raise this possibility in the discussion. The hypothesis will need to be tested experimentally.

Minor:
1. The addition of a few schematics providing an over view of how particular pathways related to ATP and energy regulation would be helpful and improve readability – HK2, HIF pathway and related genes etc.

We’ve added schematics delineating how we hypothesize HK2 knockdown and consequent glucose diversion through the PPP impact ATP consumption, and ATP production as a function of energy substrate, as supported by our new metabolomic analyses (Figure 5h).

**Reviewer comments, second version:**

Reviewer #1 (Remarks to the Author):

In the revised manuscript, the authors addressed some of my previous questions however the major concerns (see below) remain unaddressed.

Major point 1: The authors created a respiration condition by the addition of 2DG for blocking glycolysis. The rational/justification for the addition of 2-DG for inducing cellular respiration remains unclear. Additionally, the author did not verify their metabolic hits with more physiologically relevant conditions that mimic respiratory process in cells. 2-DG at a high dose at 10mM not only blocks glycolysis as expected by the authors, it would likely induce glycolysis-independent signaling pathways. This possibility cannot be rule out without verification experiments. The biological relevance of the ATP-regulatory hits identified by the CRISPR screens is thus greatly diminished.

Major point 2: Misleading justification for the inclusion of 3mM 2-DG to mimic the glycolysis condition. The authors used oligomycin to inhibit cellular respiration leading to metabolic reprogramming toward glycolysis and showed that further addition of 3mM 2-DG did not affect the oligomycin-increased glycolytic metabolites (Fig. A, in rebuttals). This result is hard to explain. The tracing experiments suggest that oligomycin induced glucose utilization in cells. Under this scenario, the cells should increase their dependency on glycolytic pathway as well as the sensitivity to 2-DG in general. The author showed that 2-DG had no effects on glycolysis inhibition is conflicting with the current knowledge. How do the authors reconcile these divergent findings


Reviewer #2 (Remarks to the Author):

The authors addressed all the points raised with the initial submission. Aspects of the paper still feel unsatisfying to me, and think the claim that hexokinase is the main consumer of ATP in the abstract is misleading. It may have been a top scoring hit in the screen, but is very unlikely to consume more ATP than protein synthesis or other processes that are not dependent on single genes that cold score. The premise of the screen is very interesting, and should be published even though many of the hits are obvious as this is a nice resource and could suggest new projects for the field.


Reviewer #3 (Remarks to the Author):

This is an excellent and thorough study. The authors adequately addressed reviewer concerns that were within the scope of the manuscript. Overall this study provides a comprehensive analysis of genes control ATP consumption and generation which is highly relevant to the broader metabolism field. The revisions to the text as well as the additional data that further investigates the role of HK2 in regulating cellular ATP levels greatly enhance the manuscript. The updated manuscript is suitable for publication in Nature Communications.

**Author rebuttal, second version:**

Reviewer #1 (Remarks to the Author):

In the revised manuscript, the authors addressed some of my previous questions however the major concerns (see below) remain unaddressed.  

Major point 1: The authors created a respiration condition by the addition of 2DG for blocking glycolysis. The rational/justification for the addition of 2-DG for inducing cellular respiration remains unclear. Additionally, the author did not verify their metabolic hits with more physiologically relevant conditions that mimic respiratory process in cells. 2-DG at a high dose at 10mM not only blocks glycolysis as expected by the authors, it would likely induce glycolysis-independent signaling pathways. This possibility cannot be rule out without verification experiments. The biological relevance of the ATP-regulatory hits identified by the CRISPR screens is thus greatly diminished.

We appreciate the reviewer’s concern. We addressed the rationale for our metabolic conditions in depth in our initial response, and so do not repeat all of the rationale and details here. Although 2DG is likely the most widely-used drug to inhibit glycolysis, we agree that all available glycolytic inhibitors can have non-specific effects. As outlined in our prior response, we believe the published effects of 2DG on prosurvival pathways referenced by the reviewer (Zhong et al., *JBC* 2009, PMID 19574224) are unlikely to have significant impact in our paradigm, due to the short incubation period with 2DG. However, we agree with the reviewer that we cannot exclude the possibility that a small number of our respiratory hits were influenced by other effects of 2DG independent of its effects on glycolysis, and have added acknowledgement of this possibility. At the same time, we also again emphasize that we did indeed achieve conditions under which cells must use respiration for ATP, and this approach identified known regulators of mitochondrial-derived ATP production. We reiterate the key points below:

1. We designed our screen to force cells to depend entirely on either respiration (respiratory conditions) or glycolysis (glycolytic conditions) for ATP, or to let cells use either. As such, the respiratory and glycolytic conditions intentionally create metabolic extremes, enabling us to identify the widest possible range of ATP regulating genes and pathways, and to provide the most robust possible data. As such, our goal was not to precisely mimic physiologic or pathologic substrates, which have generally not been defined, but this will be an important objective of future studies.

2. Our respiratory screening paradigm identified numerous components of the electron transport chain and most mitochondrial ribosomal proteins that are known to be essential for mitochondrial-derived ATP production. This proves the sensitivity of our approach to identify elements that are critical for respiration. Moreover, mutations in many of these genes cause mitochondrial disorders that are believed to produce disease through effects on energy failure, establishing the biologic and disease relevance of many of our hits. However, we agree that many of the new hits we identified will need additional investigation to determine their mechanisms of action. As we outline in the manuscript, the robust differences in how many of our hits impact ATP across different energy substrates provides initial clues into these mechanisms of action.

Major point 2: Misleading justification for the inclusion of 3mM 2-DG to mimic the glycolysis condition. The authors used oligomycin to inhibit cellular respiration leading to metabolic reprogramming toward glycolysis and showed that further addition of 3mM 2-DG did not affect the oligomycin-increased glycolytic metabolites (Fig. A, in rebuttals). This result is hard to explain. The tracing experiments suggest that oligomycin induced glucose utilization in cells. Under this scenario, the cells should increase their dependency on glycolytic pathway as well as the sensitivity to 2-DG in general. The author showed that 2-DG had no effects on glycolysis inhibition is conflicting with the current knowledge. How do the authors reconcile these divergent findings

We appreciate the reviewer’s feedback, and agree it is important to justify our use of 3mM 2DG in our glycolytic metabolic condition. To clarify, the data we provided to the reviewer is consistent with our assertions in both this submission and in our previous manuscript (Mendelsohn et al., *PLoS Biol*, PMID 30148842), where we showed that our glycolytic condition (oligomycin, 2 mM glucose and 3mM 2DG) partially and stably decreases ATP levels versus basal conditions, while also forcing all ATP to come from glycolysis. As we outline in the manuscript, this low dose of 2DG was necessary to decrease the glycolysis-derived ATP level below baseline levels (ATP levels did not acutely decrease in K562 cells with oligomycin alone in glucose), enabling us to identify high ATP genes that increase glycolytic ATP back to basal levels. Importantly, we show multiple lines of evidence supporting that both glycolysis-derived ATP production and glucose utilization continue despite the presence of this low dose of 2DG (Figure S2A in Mendelsohn et al, Figures 5c,d and S5a,b of the current manuscript, and Figure A in our prior response). Figure 3A,E from Wang et al. (*PLoS One*, 2015, PMID 26134286) also demonstrates this. The data in Figure A from our prior response indeed shows a significantly decreased metabolite pool size of F16BP, consistent with the reviewer’s expectation of increased sensitivity to 2DG. In contrast, the higher concentration of 10mM 2DG coupled with glucose withdrawal used for our respiratory conditions leads to the rapid and complete loss of glycolysis-derived ATP (Mendelsohn et al., Figs. 2b,S2A, and Fig. S5a,b from current paper). These observations are consistent with current knowledge, and 2DG’s known role as a competitive inhibitor of hexokinase (Wick et al., *JBC*, PMID 13405925).

Reviewer #2 (Remarks to the Author):

The authors addressed all the points raised with the initial submission.

Aspects of the paper still feel unsatisfying to me, and think the claim that hexokinase is the main consumer of ATP in the abstract is misleading. It may have been a top scoring hit in the screen, but is very unlikely to consume more ATP than protein synthesis or other processes that are not dependent on single genes that cold score.

The premise of the screen is very interesting, and should be published even though many of the hits are obvious as this is a nice resource and could suggest new projects for the field.

We appreciate the reviewer’s comments and suggestions, and agree this is an important distinction. HK2 was identified as the most robust consumer of ATP in our screen, but we are not claiming that HK2 consumes more energy than broad processes such as protein synthesis that involve many genes. We’ve reviewed and adjusted the language to qualify the interpretation of our result, and also added discussion that additional experiments will be required to define how HK2’s relative effects on energy consumption differ across other cell types and metabolic conditions.

Reviewer #3 (Remarks to the Author):

This is an excellent and thorough study. The authors adequately addressed reviewer concerns that were within the scope of the manuscript. Overall this study provides a comprehensive analysis of genes control ATP consumption and generation which is highly relevant to the broader metabolism field. The revisions to the text as well as the additional data that further investigates the role of HK2 in regulating cellular ATP levels greatly enhance the manuscript. The updated manuscript is suitable for publication in Nature Communications.

We appreciate the reviewer’s supportive comments and suggestions that have helped us strengthen the manuscript

**Reviewer comments, third version:**

**Author rebuttal, third version:**
